# Impact of Persistent Endocrine-Disrupting Chemicals on Human Nuclear Receptors: Insights from In Silico and Experimental Characterization

**DOI:** 10.3390/ijms26072879

**Published:** 2025-03-21

**Authors:** Harrish Ganesh, James Moran, Saptarshi Roy, Joshua Mathew, Jehosheba Ackah-Blay, Ellen Costello, Priya Shan, Sivanesan Dakshanamurthy

**Affiliations:** 1 VCU Life Sciences, Virginia Commonwealth University, Richmond, VA 22043, USA; 2 College of Arts & Sciences, Georgetown University, Washington, DC 20057, USA; 3 College of Humanities and Sciences, Virginia Commonwealth University, Richmond, VA 22043, USA; 4 University of Maryland, College Park, MD 20742, USA; 5 Cornell University, Ithaca, NY 14850, USA; 6 University of Notre Dame, Notre Dame, IN 46556, USA; 7 University of Virginia, Charlottesville, VA 22903, USA; 8 Department of Oncology, Lombardi Comprehensive Cancer Center, Georgetown University Medical Center, Washington, DC 20007, USA

**Keywords:** persistent endocrine-disrupting chemicals (EDCs), human nuclear receptors (hNRs), molecular docking simulations, PFAS, phthalates, nanoplastics, hydrophobic interactions, gene regulation, NR activation assay, NR transcription factor activation assay

## Abstract

Endocrine-disrupting chemicals (EDCs) are notable for their persistence, bioaccumulation, and associations with cancer. Human nuclear receptors (hNRs) are primary targets disrupted by these persistent EDCs, resulting in alterations to xenobiotic metabolism, lipid homeostasis, and endocrine function, which can lead to carcinogenic effects. Despite their hazardous effects, comprehensive studies on EDC interactions and their impacts on hNRs remain limited. Here, we profiled the interactions of persistent EDCs, including PFAS, plastic additives, bisphenols, polybrominated diphenyl ethers, and phthalates, with key hNRs such as PXR, CAR, PPARα, PPARγ, PPARδ, AR, and RORγt. Through controlled molecular docking simulations, we observed strong binding of the EDCs to these receptors. Further analysis showed that EDCs exhibit strong binding activity towards hNRs by preferentially interacting with hydrophobic amino acids, namely leucine, isoleucine, methionine, and phenylalanine. PFAS demonstrated the highest binding affinity, characterized by a combination of complementary hydrophobic interactions from their fluorinated carbon chains and polar interactions from their functional groups (e.g., carboxylate, sulfonate) across all receptors. Distinct polycyclic and hydrophobic trends, contributing to strong NR binding, were evident in non-PFAS and nonplastic EDCs. The hNR activity assay in HepG2 cells revealed agonistic effects of dicyclohexyl phthalate (DCHP) and di-2-ethylhexyl phthalate (DEHP) on most receptors, except for PPARα. The hNR transcription factor pathway assay in HepG2 cells demonstrated increased gene expression of VDRE and PXR, suggesting potential chronic effects on xenobiotic metabolism and calcium homeostasis. Overall, our findings demonstrate the need for further research into the endocrine disruption and carcinogenic effects of these persistent EDCs.

## 1. Introduction

Persistent EDCs, such as PFAS, are a class of synthetic chemicals that have been used widely for decades and are now ubiquitous in the environment as they have been incorporated into a variety of industrial and consumer products [1]. PFAS have been found to be exposed to nearly all individuals in the United States [2]. PFAS have revealed adverse health effects such as cancer, metabolic disorders, and reproductive issues [3]. Other EDCs, such as polybrominated diphenyl ethers (PBDEs), phthalates, PFAS pesticides, bisphenols, and nanoplastics, are emerging concerns. PBDEs are known to bioaccumulate, potentially causing endocrine disruption, developmental and neurological impairments, and cancer [4]. Altogether, these EDCs commonly co-occur in the environment and are co-exposed as a mixture, which exacerbates the adverse health effects [5]. PBDEs, common flame retardants, are chemically stable and persist in the environment [6]. Phthalates, used as plasticizers, interfere with hormonal signals that lead to reproductive and developmental abnormalities, metabolic issues, and increased risk of cancer [7]. Phthalates have also been shown to act as EDCs [7]. Bisphenol compounds have been shown to display endocrine-disrupting functions in addition to metabolic disease when co-exposed with other EDCs [8]. PFAS pesticides are harmful due to their environmental persistence and resistance to degradation; they have been shown to have endocrine-disrupting effects, and are correlated with liver damage, immune system dysfunction, and developmental issues [9,10]. Nanoplastics, while less toxic by themselves, become a major concern when they carry hazardous chemicals like PBDEs, phthalates, bisphenols, and PFAS on their surfaces [11]. Nanoplastics, widespread in aquatic environments, can introduce toxic substances into the food chain when ingested by marine and terrestrial organisms [12]. Plastic chemicals have the ability to disrupt human endocrine function when consumed in nanomolar amounts [13]. This combined presence of contaminants creates hazards by affecting ecosystems and human health [14,15]. Furthermore, a comprehensive understanding of the interaction of these pollutants is crucial to addressing their endocrine-disrupting effects and their impact on health.

EDCs have been shown to activate nuclear receptors. For example, human and rat sample analyses have demonstrated that PFAS activates peroxisome proliferator-activated receptors (PPARs), particularly PPAR alpha (PPARα) and PPAR gamma (PPARγ). Common PFAS, including perfluorooctanoic acid (PFOA), perfluorooctanesulfonic acid (PFOS), and perfluorohexane sulfonic acid (PFHxS), frequently activate these receptors [16]. Xie et al. correlate PFAS exposure with the activation of PPARδ and PPARγ, with PPARα displaying greater activation in comparison to other isoforms [17]. Perfluorinated carboxylic acids (PFCAs), particularly PFOA, indirectly activate the constitutive androstane receptor (CAR) in mouse and human models, emphasizing the investigation of PFAS interactions with nuclear receptors [18]. Perfluorohexane sulfonate (PFHxS) exposure has been shown to affect important regulatory cell-cycle proteins in part through the activation of CAR [19]. Cell toxicity has been linked to pregnane X receptor (PXR) activation following exposure to PFOA and PFOS, displaying harmful downstream effects of PFAS [20]. Also, PFAS have demonstrated antagonistic effects on the androgen receptor (AR) as competitive inhibitors of testosterone, disrupting normal hormonal functions [21]. PFOS exposure has been associated with an increase in Retinoic Acid-Related Orphan Receptor γt+ (RORγt+) CD4+ T cells, indicating disruption of the immune system [22].

Although EDCs can individually activate NRs, co-exposure to different EDC classes can also lead to agonistic or antagonistic effects on nuclear receptors. Rickard et al. demonstrated that co-exposure to PFAS and phthalates can create disruptions in hormonal signaling, leading to more adverse health effects [3]. Limbu et al. reported the carcinogenic potential of various co-exposed EDC mixtures [23]. Wu et al. reported that co-exposure to various PBDEs causes endocrine disruption and neurotoxicity [24]. Further, nanoplastics act as carriers for various EDC classes including PFAS, phthalates, and bisphenols, showing the importance of studying the binding potential of nanoplastics and EDCs [25,26,27,28]. Additionally, Deng et al. demonstrated that co-exposure to nanoplastics and phthalates resulted in increased intestinal inflammation, demonstrating the consequences of co-exposure [26]. The bioaccumulation and consistent exposure of these substances justify evaluating their binding potential to seven key nuclear receptors [4,29,30,31].

PPARα, PPARγ, PPARδ, PXR, RORγt, AR, and CAR are major regulators of gene expression related to metabolism, detoxification, and cell proliferation. PPARα, PPARγ, and PPARδ are relevant in fatty acid metabolism, energy homeostasis, and inflammation, and they significantly influence lipid metabolism and insulin sensitivity [32]. PXR and CAR are regulators of xenobiotic metabolism, as they are involved in eliminating foreign substances, inducing detoxification enzymes, and influencing energy metabolism and liver function [33]. RORγt is essential in modulating immune responses through its role in Th17 cell differentiation and function, as well as in the development of lymphoid tissues [34]. AR is widely expressed and plays diverse roles across the reproductive, musculoskeletal, cardiovascular, immune, neural, and hematopoietic systems, and is involved in tumor development in various organs [35]. These receptors form heterodimers with each other, allowing them to interact with various EDCs, both individually and in mixtures [36,37,38]. Taken together, despite the bioaccumulation potential, constant exposure, interaction with hNRs, and hazardous health effects of EDCs, comprehensive studies on these persistent EDC interactions and their impacts on hNRs remain limited.

The primary goal of this study is to analyze the binding interactions and strength of persistent EDCs, including PFAS, plastic chemicals, pesticides, bisphenols, polybrominated diphenyl ethers, and phthalates, with human nuclear receptors (hNRs) such as PXR, CAR, RORγt, AR, PPARα, PPARγ, and PPARδ. A secondary goal is to explore the physicochemical properties and structural features of EDCs that influence their binding strength with these receptors. We also examined the physicochemical and structural characteristics of PFAS to understand their role in binding affinity. This study represents the first comprehensive analysis of interactions across six EDC chemical classes with the hNRs and identified common characteristics that contribute to effective binding. Further, our results displayed a range of EDCs with strong binding affinity, as demonstrated through in vitro hNR activation and transcription factor assays, underlining the need for further investigation. We also provide the first analysis of a dataset comprising 16,892 EDCs, many of which have not been extensively studied before. By simulating molecular binding interactions between these toxic substances and NRs, this work reveals new insights into the structural and functional attributes of EDCs that drive binding affinity. Furthermore, we developed automated, user-friendly Python 3.12.4 programs to assess amino acid residue interactions and the influence of functional groups on binding strength.

## 2. Results and Discussion

### 2.1. Validation of Docking Protocol

**Figure 1:**
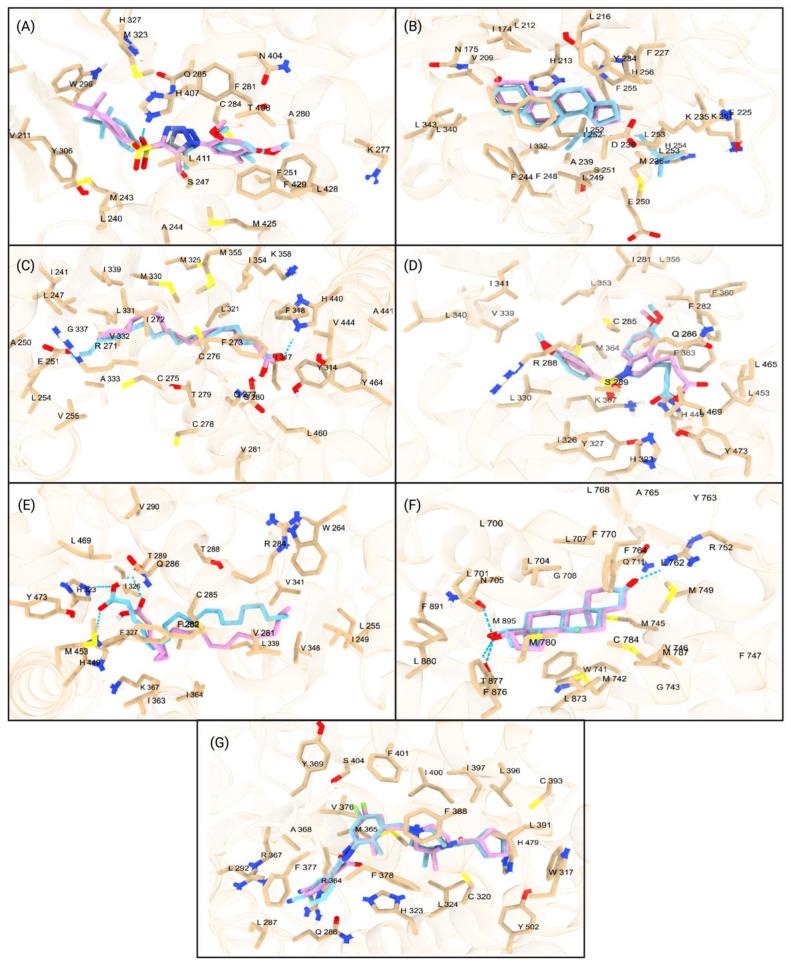
(**A**) Superimposed PXR structure in brown (PBD ID: 5X0R) and docked binding pose of PXR orthosteric ligand 4WH. The reference binding pose of SJB7 is shown in blue. The redocked endogenous ligand (pink), SJB7, was superimposed on the reference ligand (cyan) with an RMSD value of 1.720 Å. (**B**) Superimposed CAR co-crystal structure in blue (PBD ID: 1XNX) and docked binding pose of androstenol (ATE) in pink. The reference binding pose of ATE is shown in blue. (**C**) Redocked palmitic acid (pink) with a score of −6.4 kcal/mol superimposed on the native position (blue) at the orthosteric site of the PPARα LBD. The root-mean-square deviation (RMSD) was calculated to be 1.495 Å, indicating the accuracy of the docking protocol. (**D**) Superimposed depiction of redocked ET1 ligand (pink) onto co-crystallized ET1 ligand (blue) displayed (PDB ID: 3ET3) at the orthosteric site of PPARγ is shown, with an RMSD of 1.060 Å. (**E**) The endogenous ligand vaccenic acid (cyan) was redocked at the PPARδ LBD. The structural alignment with the co-crystallized ligand (pink) revealed an RMSD value of 2.416 Å. (**F**) Androgen receptor with redocked DHT (pink) and native co-crystallized DHT (blue) (PDB ID: 2PIV). The RMSD value between the redocked and endogenous confirmation of DHT is 0.353. (**G**) Superimposed conformation of the redocked Ursolic Acid (pink) and the co-crystalized structure of Ursolic Acid (blue) (PDB ID: 6O3Z) on the RORγt protein. The structural alignment revealed an RMSD value of 0.822. All amino acid residues within 5 Å of the redocked ligand are shown. All hydrogen bonds are represented with a blue dotted line. Specific ligands and experimental binding affinity and predicted binding affinities can be found in Supplementary Table S10.

The molecular docking protocol was validated by assessing Autodock Vina accuracy in predicting the binding poses of reference ligands. For PXR, the agonist SJB7 yielded an RMSD of 1.720 Å (Figure 1A). The predicted binding conformation of 16,17-Androstene-3-ol (ATE), bound to CAR, was compared to its co-crystal structure (Figure 1B), resulting in an RMSD value of 0.709 Å. Palmitic acid, an endogenous ligand of PPARα, was docked and compared to its co-crystal structure, with an RMSD of 1.495 Å (Figure 1C). ET1, an endogenous ligand of PPARγ, had an RMSD of 1.060 Å (Figure 1D). For PPARδ, the binding pose of the endogenous ligand vaccenic acid was compared to the co-crystal structure, resulting in an RMSD of 2.416 Å (Figure 1E). The binding pose of dihydrotestosterone (DHT) with AR yielded an RMSD of 0.353 Å (Figure 1F). Finally, the binding pose of the RORγt with LKY was compared to the co-crystal structure, resulting in an RMSD of 0.822 Å (Figure 1G). The RMSD values below 2.5 Å confirm the accuracy of this study in predicting the binding poses of ligands to the studied NRs. Then, the predicted docking scores of known ligands were compared to known IC50/EC50 values to validate the accuracy of Autodock Vina. The ligands of CAR, PXR, PPARα, PPARδ, PPARγ, AR, and RORγt were docked, and the binding affinities were compared with experimentally determined IC50/EC50 values, resulting in standard errors of 0.82, 0.54, 0.389, 1.39, 0.34, 0.25, and 1.44, respectively. The low standard error of our data validates the accuracy of Autodock Vina in predicting the binding affinity of ligands bound to various NRs.

### 2.2. Binding Strength Analysis

#### 2.2.1. PFAS Binding Strength Analysis

The binding strength analysis of PFAS bound to PXR revealed that a subset of them displayed strong binding interactions (Table 1), while a smaller portion showed strong interactions with CAR (Table 1). This potential receptor activation is significant because CAR and PXR are involved in regulating xenobiotic and drug metabolism, as well as detoxification processes. Activation of these receptors by PFAS may disrupt metabolic functions, potentially leading to toxicological effects [31]. Further analysis showed that 13.9% of PFAS demonstrated moderate binding affinity to CAR, and 16.8% to PXR, with most showing weak binding to both. Analysis of PPAR isoforms revealed that a small subset of PFAS displayed strong binding interactions with PPARα (Table 1), a higher proportion with PPARγ (Table 1), and a smaller portion with PPARδ (Table 1). These findings emphasize that certain PFAS have the potential to activate PPARs, particularly PPARγ, as demonstrated by their strong binding affinities, consistent with the previous literature [16]. A total of 11.4% of PFAS were moderate binders to PPARα, 19.0% to PPARγ, and 12.0% to PPARδ. Most PFAS showed weak binding across the PPAR isoforms. Activity of PPARs by PFAS may contribute to disruptions in lipid metabolism, energy homeostasis, and inflammation pathways, leading to metabolic disorders, obesity, and other health issues [17]. PFAS interactions with AR displayed a minimal subset of compounds as strong binders, indicating limited potential for AR activation (Table 1), while a larger subset of PFAS showed strong binding affinity for RORγt (Table 1). Activation by PFAS can disrupt AR and RORγt, potentially affecting male reproductive development and immune response regulation, respectively [34,35]. A total of 2.1% of PFAS showed moderate binding affinity to AR, and 13.5% have moderate binding affinity to RORγt. Most PFAS showed weak binding to both AR and RORγt, with AR interactions being particularly limited, as detailed by the raw binding affinities provided in Supplementary Table S3.

#### 2.2.2. Plastic Chemical Binding Strength Analysis

**Figure 2:**
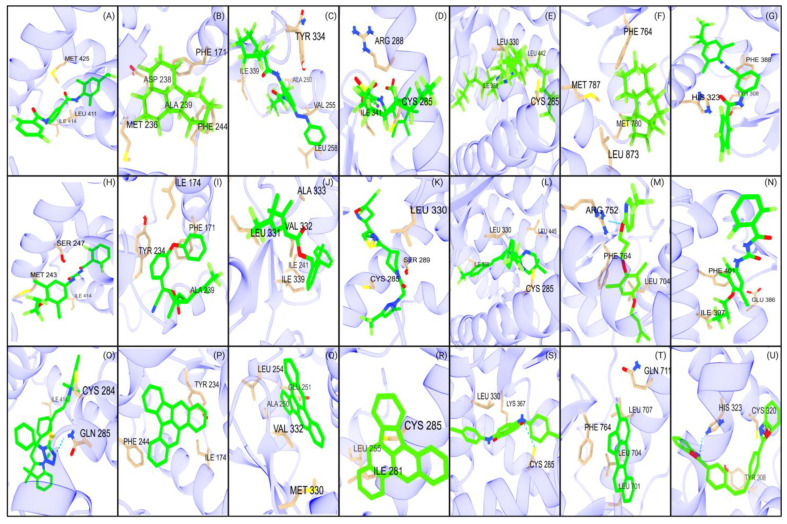
Major interactions of studied NRs with PFAS, PFAS pesticides, and nanoplastics. The first row (**A**–**G**) represents the interactions of seven NRs with PFAS: (**A**) PXR, (**B**) CAR, (**C**) PPARα, (**D**) PPARγ, (**E**) PPARδ, (**F**) AR, and (**G**) RORγt. The second row (**H**–**N**) illustrates the interactions of the same NRs with PFAS pesticides: (**H**) PXR, (**I**) CAR, (**J**) PPARα, (**K**) PPARγ, (**L**) PPARδ, (**M**) AR, and (**N**) RORγt. The third row (**O**–**U**) depicts the interactions of these NRs with nanoplastics: (**O**) PXR, (**P**) CAR, (**Q**) PPARα, (**R**) PPARγ, (**S**) PPARδ, (**T**) AR, and (**U**) RORγt. Hydrogen bonds are represented with a blue dotted line.

Nanoplastics were classified as strong binders to PXR, with 6.5% demonstrating strong binding affinity (Table 2), and 1.5% demonstrating strong binding affinity to CAR (Table 2), suggesting that these ligands are potential activators of these nuclear receptors. In contrast, the majority of the nanoplastics were classified as weak binders. Strong binders to PPAR isoforms represent a small fraction of the total nanoplastics tested for PPARα (Table 2), PPARγ (Table 2), and PPARδ (Table 2). For PPARα, a minor portion of the nanoplastics displayed strong and moderate binding affinities, while a larger proportion showed similar binding to PPARγ and PPARδ. The high presence of moderate and strong PPARγ binders suggests an increased likelihood of nanoplastic interactions that may lead to obesity and irregular adipose homeostasis [39]. The structural similarity of certain plastic chemicals to endogenous ligands allows for the upregulation of adipogenesis through activation of these NRs [39]. Despite this, a substantial portion of the nanoplastics were classified as weak binders (Table 2). Nanoplastic 3-methylcholanthrene (CAS ID: 56-49-5) was one among the total tested, showing strong interaction with AR (Table 2). In contrast, many nanoplastics demonstrated strong binding affinity to RORγt (Table 2), suggesting a higher potential for receptor activation compared to AR. Few nanoplastics displayed moderate or strong binding with AR, while a larger portion showed stronger interactions with RORγt, as shown in Supplementary Table S4. Overall, most nanoplastics exhibited weak binding to both receptors, with interactions with AR being weaker than those with RORγt. Nanoplastics generally contain small proportions of strong to moderate binders across all NRs. The plastic chemical (CAS ID: 5385-75-1) emerged as the top binder for CAR, PPARα, and PPARγ, showing the highest binding affinity with CAR (−12.1 kcal/mol). Its heterocyclic structure enables stabilization within the hydrophobic pockets of these NRs (Figure 2P–R). The strongest overall binder (CAS ID: 128-80-3) demonstrated a binding affinity of −12.6 kcal/mol with PPARδ (Figure 2S), due to the hydrogen-bonding and cyclic characteristics in the hydrophobic pocket. These findings suggest that while nanoplastics may have a relatively lower potential for NR activation compared to PFAS, the cyclic nature of certain nanoplastics can still enable NR activation.

#### 2.2.3. Phthalate Binding Strength Analysis

**Figure 3:**
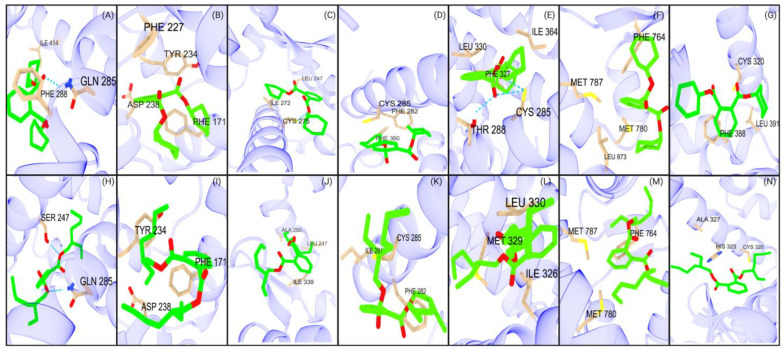
Major interactions of studied NRs with DCHP and DEHP. The first row (**A**–**G**) represents the interactions of seven NRs with DCHP: (**A**) PXR, (**B**) CAR, (**C**) PPARα, (**D**) PPARγ, (**E**) PPARδ, (**F**) AR, and (**G**) RORγt. The second row (**H**–**N**) illustrates the interactions of the same NRs with DEHP: (**H**) PXR, (**I**) CAR, (**J**) PPARα, (**K**) PPARγ, (**L**) PPARδ, (**M**) AR, and (**N**) RORγt.

CAR and PXR each identified a single moderate binder among the interacting phthalates (Supplementary Figure S1A,B). PPARγ’s ligand composition included both strong and moderate binders, while PPARδ included a small set of moderate binders (Supplementary Figure S1D,E). PPARα displayed exclusively weak binders (Supplementary Figure S1C), with PPARγ and PPARδ predominantly showing weak binding. Similarly, AR and RORγt receptors displayed only weak binders, except for one moderate binder identified in RORγt (Supplementary Figure S1F,G). These findings suggest that certain phthalates have lower binding potential relative to other compound classes, such as PFAS and nanoplastics, as demonstrated in Supplementary Tables S3–S5. However, specific phthalates, such as DCHP and DEHP, showed stronger binding to NRs. DCHP consistently displayed the strongest interactions between NRs among all phthalates tested. DCHP interacting with PPARδ had the highest binding affinity of −9.6 kcal/mol due to hydrogen-bonding and van der Waals interactions (Figure 3E). Similarly, PXR and PPARα displayed hydrogen bonding, with binding affinities of −9.0 kcal/mol and −8.6 kcal/mol, respectively (Figure 3A,C). In addition to hydrogen bonding stabilization, cysteine plays a major role in nanoplastic interactions across many receptors (Figure 3A). Therefore, DCHP and DEHP can be considered as potential endocrine disruptors.

#### 2.2.4. PBDE Binding Strength Analysis

The results were uniform, with CAR, PXR, PPARα, PPARδ, RORγt, and AR showing nearly identical binding profiles (Supplementary Figure S2, Supplementary Table S6). Across these receptors, 100% of the interacting ligands were weak binders. PPARγ, however, displayed a slightly different profile, with 98.6% weak binders and 1.4% moderate binders (Supplementary Figure S2D). This indicates that PBDEs have a minimal capacity to induce receptor activation.

#### 2.2.5. Bisphenol Binding Strength Analysis

**Figure 4:**
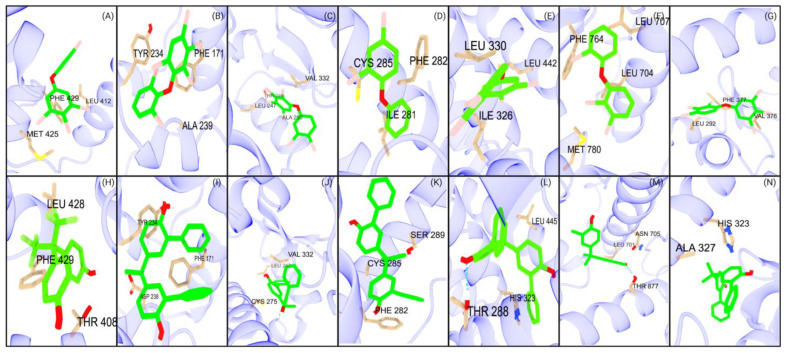
Top PBDE and BPA major interactions with studied NRs. The first row (**A**–**G**) illustrates the interactions of the seven NRs with the top PBDEs: (**A**) PXR, (**B**) CAR, (**C**) PPARα, (**D**) PPARγ, (**E**) PPARδ, (**F**) AR, and (**G**) RORγt. The second row (**H**–**N**) depicts the interactions of the same seven NRs with the top BPAs: (**H**) PXR, (**I**) CAR, (**J**) PPARα, (**K**) PPARγ, (**L**) PPARδ, (**M**) AR, and (**N**) RORγt.

With the greatest number of strong binders, PXR and CAR demonstrated the strongest binding affinities among the receptors (Supplementary Figure S3A,B). Additionally, PXR showed 21.7% moderate binders, while CAR had 5.3% moderate binders. Among the PPAR isoforms, both PPARα and PPARδ had a limited number of strong binders, whereas PPARγ displayed a comparatively higher binding affinity, with a greater number of strong and moderate binders (Supplementary Figure S3C–E). In contrast, only RORγt exhibited a moderate binder among AR and RORγt, with all other ligands for the receptors classified as weak binders (Supplementary Figure S3F,G). The structural analyses reveal that bisphenol (CAS ID: 24038-68-4) has the strongest binding interactions with CAR, PPARα, PPARγ, and RORγt (Figure 4I–K,N), with raw binding affinities provided in Supplementary Table S7. Bisphenols show the highest affinity with CAR (−11.5 kcal/mol), with polar residues such as asparagine and histidine contributing significantly to their stability. Bisphenols are potentially carcinogenic, as they tend to bind more strongly than PBDEs but more weakly than other compound classes such as PFAS.

#### 2.2.6. PFAS Pesticide Binding Strength Analysis

PFAS pesticides displayed variable binding interactions with different nuclear receptors (Supplementary Table S8). PXR showed the highest number of strong binding interactions, with 43.2% strong binders and 21.6% moderate binders (Supplementary Figure S4). In contrast, CAR had 8.1% moderate binders, with the remainder classified as weak binders (Supplementary Figure S4B). PPARα, PPARγ, and PPARδ displayed 2.6%, 47.4%, and 23.7% strong binders, respectively (Supplementary Figure S4C–E). The number of moderately binding ligands was more consistent, with 28.9%, 26.3%, and 18.4% for PPARα, PPARγ, and PPARδ, respectively. RORγt has 10.5% strong binders and 31.6% moderate binders, while AR exhibited only weak binding interactions (Supplementary Figure S4F,G). This pattern of variable binding interactions persisted across other nuclear receptors. PXR had the highest number of strong binding interactions (Supplementary Figure S4). PPARα, PPARγ, and PPARδ showed some strong binding interactions, with PPARγ displaying the highest number of strong binders, followed by PPARδ and PPARα (Supplementary Figure S4C–E). Moderately binding ligands were consistently observed across the three PPAR isoforms. RORγt included a subset of strong binders and a considerable portion of moderate binders, while AR exhibited only weak binding interactions (Supplementary Figure S4F,G). The structural analyses reveal that PFAS pesticide Hexaflumuron (CAS ID: 86479-06-3) shows the strongest binding interactions with PXR (Figure 2O). PFAS pesticides show the highest affinity with CAR (−11.5 kcal/mol), with nonpolar residues like leucine, isoleucine, and phenylalanine contributing significantly to their stability. Overall, PFAS pesticides showed varying binding affinities with nuclear receptors, ranging from strong interactions to exclusively weak binding with AR.

### 2.3. Amino Acid Interaction Analysis of EDC Classes

**Figure 5:**
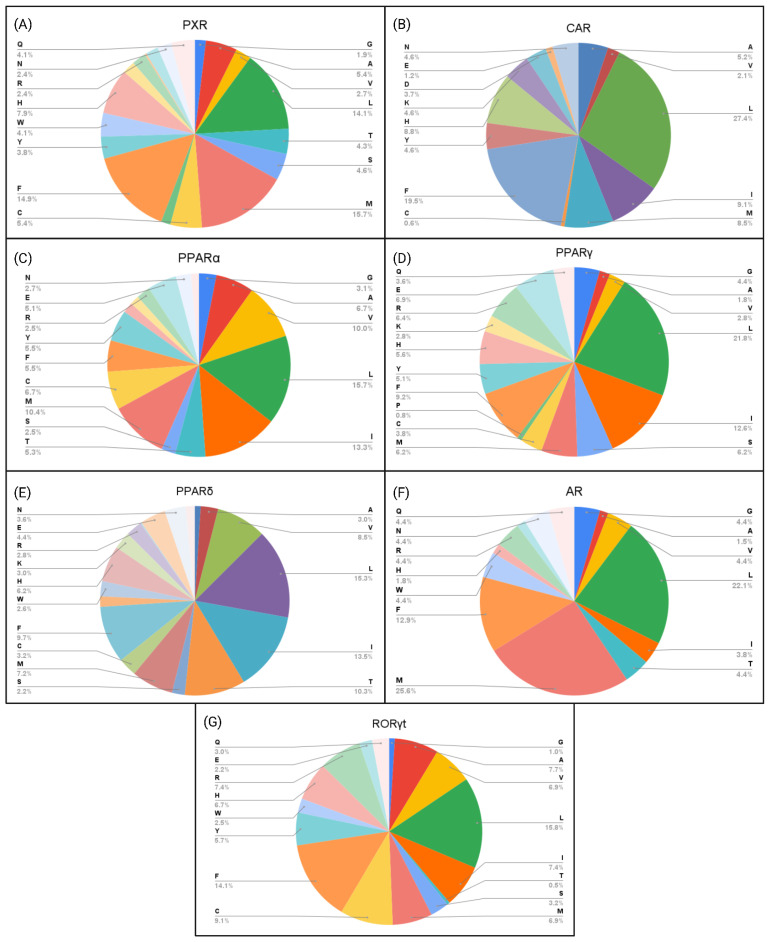
Percentage of NR amino acid residues interacting with the top 15 PFAS binders. (**A**) PXR, (**B**) CAR, (**C**) PPARα, (**D**) PPARγ, (**E**) PPARδ, (**F**) AR, and (**G**) RORγt.

**Figure 6:**
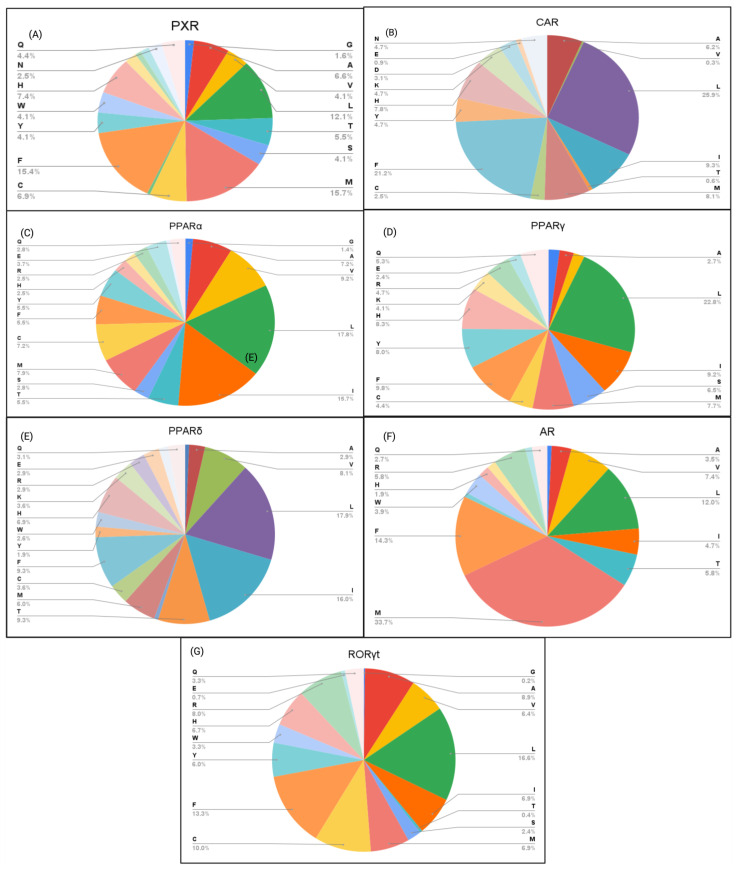
Percentage of NR amino acid residues interacting with the top 15 nanoplastic binders. (**A**) PXR, (**B**) CAR, (**C**) PPARα, (**D**) PPARγ, (**E**) PPARδ, (**F**) AR, and (**G**) RORγt.

Next, we investigated how key interacting amino acid residues correlate with the hydrophobic and polar characteristics of EDCs, the impact of these characteristics on the predicted binding affinity (Figure 2, Figure 3, Figure 4, Figure 5 and Figure 6), and their correlation with the interacting amino acid frequencies (Supplementary Figure S5). Among the top PFAS binders, the predominant residues were leucine, phenylalanine, and isoleucine for CAR (Figure 5B), while leucine, methionine, phenylalanine, and histidine were common within PXR (Figure 5A). The top three amino acids with the most interactions for both CAR and PXR were hydrophobic, which likely creates an optimal environment for PFAS binding. The nonpolar characteristics of PFAS molecules complement the hydrophobic binding pockets of the receptors, resulting in stronger and more specific interactions. This complementarity shows the importance of hydrophobic interactions in the binding of PFAS to the receptors. However, polar interactions also play important roles in PFAS binding with receptor LBDs. For example, the PFAS (CAS ID: 662-28-2) with the highest binding affinity among all NRs binds to CAR through interactions between its fluorine residues and HIS213 and ASN175 (Figure 2B). These polar amino acids feature the role of dipole–dipole interactions in stabilizing PFAS–receptor binding.

Within PPARα, the key interacting residues with EDCs were leucine, isoleucine, and methionine, with leucine having the highest frequency of interaction (Figure 5C). The LBD of PPARα, along with the central region of the protein, is essential for the binding of endogenous fatty acids, which have long hydrophobic tails [40,41]. Unlike other EDCs, bisphenols interacted with leucine and methionine at equal frequencies, with valine being the next frequent interaction (Supplementary Figure S6). For PPARγ, leucine, isoleucine, and phenylalanine were the key amino acid residues interacting with all EDC classes (Figure 2K,R and Figure 5D). The hydrophobic residues are consistent with the presence of a large hydrophobic pocket in the PPARγ [42,43]. Nanoplastics, PFAS, and PFAS pesticides interacted more with the hydrophilic residues arginine and glutamic acid than phthalates, PBDEs, and bisphenols (Figure 4, Figure 5 and Figure 6, Supplementary Figures S5–S14). The amino acid residues interacting with PPARδ primarily involve leucine, isoleucine, valine, and threonine (Figure 5E). These amino acids have a hydrophobic character aside from threonine, which is consistent with the hydrophobic cleft on PPARδ [41]. These residues are seen to interact with all EDC classes, reinforcing the strong hydrophobic interaction capacity between EDCs and PPAR NRs. The amino acid interactions between AR and EDCs primarily consisted of leucine, methionine, and phenylalanine (Figure 5F). These amino acids, known for their strong hydrophobic nature, play a key role in hydrophobic interactions within the AR LBD [44]. Similarly, RORγt reveals that the top two interacting amino acid residues were phenylalanine and leucine (Figure 5G), which aligns with the hydrophobic nature of RORγt LBD [45]. Raw amino acid frequency data for PFAS and nanoplastic binding interactions are displayed in Supplementary Figures S1 and S2. Overall, the results show the importance of hydrophobic residues in nuclear receptors for effective EDC binding.

### 2.4. Structural Analysis

#### Structural Analysis of PFAS

**Figure 7:**
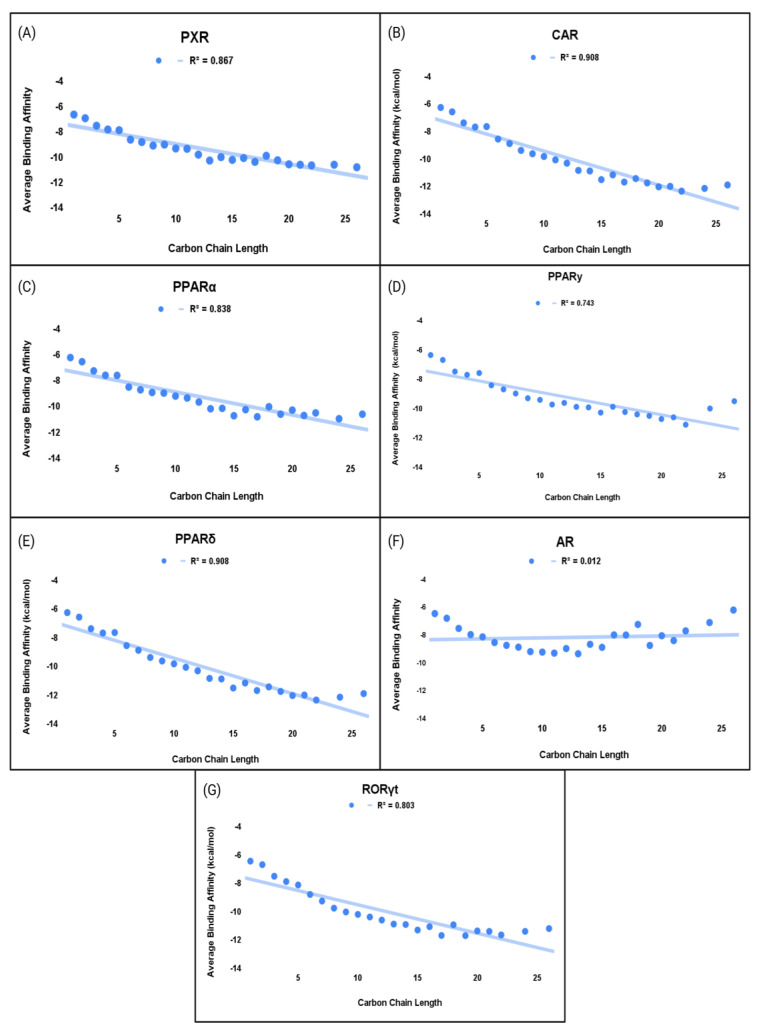
Comprehensive chain length vs. average binding affinity of PFAS to NRs. (**A**) PXR, (**B**) CAR, (**C**) PPARα, (**D**) PPARγ, (**E**) PPARδ, (**F**) AR, and (**G**) RORγt.

The polycyclic structure of PFAS plays a strong role in their binding affinities to NRs. The presence of a polycyclic form of the EDCs allows for stronger hydrophobic interactions in LBD, as seen in CAR and PPARγ. Our data show that longer, fluorinated carbon chains also strengthen the binding interactions between PFAS and NRs (Figure 7). Carboxylic acid and sulfonic acid groups of PFAS were observed to form hydrogen bonds (Figure 2). Additionally, the highly electronegative nature of fluorine in carbon–fluorine bonds increases its propensity for hydrogen bonding. For example, serine residues in the NRs participate in weak hydrogen bonding with fluorine, and interactions with oxygen are also observed (Figure 2) [46,47]. Further, van der Waals forces also contribute to these interactions [48]. These findings showed the diverse nature of dipole–dipole and hydrogen-bonding interactions between PFAS and NRs. Among the studied NRs, the PFAS compound (CAS ID: 662-28-2) demonstrated the strongest interaction with CAR, showing a binding affinity of −17.1 kcal/mol (Figure 2B). This compound, a heterocycle containing three hexyl and one pentyl ring, structurally mimics androstanol, the endogenous ligand of CAR [49]. The high binding affinity can be attributed to its structural similarity to the endogenous ligand and its hydrophobic characteristics, which allow for a stronger interaction compared to other PFAS compounds.

The electronegativity of fluorine atoms enables weak amino acid residues that contribute to the stability and high binding affinities of PFAS within the receptors. The top PFAS binding to PPARα (CAS ID: 548470-06-0) has a central aromatic ring linked to two fluorinated carbon rings through an amide group, and to an additional aromatic ring via an azo bond. This tricyclic structure was highly prevalent among the top-binding PFAS. Aromatic rings strengthen the binding affinity of PFAS by increasing hydrophobic interactions [33]. Therefore, PFAS containing multiple aromatic rings have the potential to be highly toxic, as indicated by the stronger binding affinity of these PFAS to PPARα. Similarly, the top PFAS bound to PPARγ (CAS ID: 57589-85-2) consisted of two aromatic rings in addition to an extended fluoro-carbon chain connected via a sulfone group.

Structural analyses revealed a prevalence of polycyclic forms, such as the 662-28-2 and the tricyclic 306-91-2, which contributed to the stability of these compounds by enabling interactions with the hydrophobic binding pocket of CAR. The presence of multiple rings and fluorinated carbons increases the surface area and volume of these compounds, increasing the hydrophobic contacts between PFAS and CAR. An analysis of top-binding PFAS with PPARγ shows a comparable trend, where polycyclic rings contribute significantly to strong binding interactions. Four compounds, CAS ID: 919293-77-9, 646533-88-2, 919293-78-0, and 919293-76-8, contain five carbon rings, contributing to their high binding affinity. PFAS structural characteristics studied by Roy et al. presented similar data to the aforementioned results [50]. The length of fluorinated carbon chains plays an important role in PFAS binding affinity to the PPARγ LBD. A strong correlation (R^2^ = 0.74) exists between fluorinated carbon chain length and average docking scores (Figure 7). The highest binding affinity occurs at a carbon chain length of 22 (−11.1 kcal/mol) and the lowest at 1 (−6.4 kcal/mol), but the relationship is not strictly linear. Intermediate carbon chain lengths sometimes show binding affinities that deviate from the general trend. Almeida et al. suggest that this variability may be due to functional groups, such as sulfonic and carboxylic groups, in addition to carbon chain length [51]. However, the data do not show a significant correlation between binding affinity, carbon chain length, and functional group presence (Table 3). Figure 7 illustrates the distribution of carbon chain lengths, categorizing them into short chains (carbon chain lengths from 1 to 6), long chains (carbon chain lengths of 7 and above), and a comprehensive chain length (from 1 to 26). Overall, the data reveal a general trend where longer carbon chains show higher binding affinities across all nuclear receptors compared to shorter-chain PFAS.

Almeida et al. showed that PFAS with longer carbon chain lengths generally have higher binding affinities for the PPARγ LBD [51]. Longer fluorinated carbon chains can form hydrogen bonds with Tyr473, a key amino acid in PPARγ activation [52]. Furthermore, longer carbon chains offer greater conformational flexibility, allowing the molecule to adopt a favorable binding conformation within the receptor’s LBD. Analysis of the carbon chain length of PFAS and their binding affinities to PPARδ and PPARα shows results similar to those observed with PPARγ. For PPARα, the highest binding affinity occurs at a carbon chain length of 24, with a free energy value of −11 kcal/mol, while the lowest occurs at a length of 1, with a value of −6.2 kcal/mol. For PPARδ, the highest affinity is at a length of 22, with a free energy value of −12.4 kcal/mol. The R^2^ values for the correlation between carbon chain length and average binding affinity for PPARδ and PPARα are 0.908 and 0.838, respectively (Figure 7). The high R^2^ values, along with the similar carbon chain lengths for the highest and lowest binding affinities across all PPARs, demonstrate a strong consistency between chain length and binding affinity. In addition to the PPARs, PXR also showed a strong relationship between carbon chain length, with an R^2^ value of 0.867 (Figure 7). The highest binding affinity, averaging −11 kcal/mol, was observed with a carbon chain length of 24, while the lowest, averaging −6.2 kcal/mol, occurred at a chain length of 1. The similarity in results between PXR and the PPARs can be seen due to the comparable LBD sizes, with PXR’s LBD ranging from 1290 to 1540 Å^3^, and PPARs’ LBDs ranging between 1300 and 1400 Å^3^ [53,54]. Therefore, the larger LBD of these receptors allows for more residues to interact with longer-chain PFAS.

For AR and CAR, the trend differs from other nuclear receptors. A weaker correlation is seen between carbon chain length and binding affinity. The raw data for short-chain and long-chain carbon chain analysis can be found in Supplementary Figures S15 and S16. The R^2^ value for both AR and CAR was less than 0.05, indicating that carbon chain length has minimal impact on binding affinity for these receptors (Figure 7). For AR, the highest binding affinity was correlated with a carbon chain length of 13, with an average binding affinity of −9.3 kcal/mol, and the lowest at a length of 26, with an average binding affinity of −6.2 kcal/mol. For CAR, chain lengths of 13 and 15 revealed the greatest binding affinity of −11.1 kcal/mol and the lowest at 1, with an average binding affinity of −6.5 kcal/mol. This lack of correlation between carbon chain length and binding affinity may be due to the smaller and more rigid ligand-binding domains of AR and CAR, which limit their ability to accommodate longer fluorinated carbon chains compared to receptors like PXR and PPARs [53,54]. This may also be affected by the lack of available amino acid residues to interact with the EDCs. The size of AR’s LBD was calculated to be 582 Å^3^, and 600 Å^3^ for CAR, further proving the limited capacity of these two NRs to accommodate longer-chain PFAS molecules [44,55].

Overall, fluorine atoms contributed to strong interactions between the NRs and PFAS, allowing for hydrogen bonding with key amino acid residues. Fluorine atoms participated in unique hydrogen bonding due to their high electronegativity. These interactions were likely affected by the partial positive charges on the hydrogen atoms of the amino acid side chains, which were attracted to the partial negative charges on the fluorine atoms. This hydrogen bonding enhances the binding affinity and stability of PFAS within the binding pocket of the NRs. Asn175 and His213 were essential for CAR affinity, with the amide side chain of Asn175 and the imidazole ring of His213 forming hydrogen bonds with the electronegative fluorine atoms of PFAS. The involvement of Asn175 and His213 highlights the complexity of the binding mechanism and fluorine’s role in mediating both hydrophobic and polar interactions, contributing to the high docking scores of these compounds.

### 2.5. Hydrophobicity Analysis

#### 2.5.1. Hydrophobicity Analysis of PFAS

Log*p* values, which are indicative of a compound’s partition coefficient between octanol and water, provided useful insights into the hydrophobicity of PFAS, as well as their bioaccumulation potential. All top PFAS binders to CAR and PXR displayed high predicted Log*p* values. For CAR, these values ranged from 5.94 to 9.11, while for PXR, they ranged from 6.15 to 15.32. The consistently high Log*p* values across these top binders illustrate the importance of hydrophobic interactions in the binding strength of PFAS to both receptors. In the case of CAR, the absence of significant polar surface area values among these top binders reinforced their hydrophobic profile and indicated a lack of polar functional groups. The presence of significant polar surface area values among the top PXR binders suggested that polar functional groups were also important for interaction with the PXR ligand-binding domain, which could form hydrogen bonds with PXR residues such as Gln285. This illustrated the importance of both hydrophobic and polar interactions in the binding of PFAS to PXR [56]. Similarly to PXR, PPARγ shows the importance of both hydrophobic and polar interactions in the binding of PFAS, as shown by the presence of polar functional groups such as sulfones, ketones, ethers, and amides (Supplementary Table S3). For PPARγ, the Log*p* values ranged from 4.96 to 13.7 among the top 15 PFAS binders. For RORγt, the Log*p* values ranged from 6 to 13.5, following a very similar trend as PXR. For RORγt, both hydrophobic and polar interactions were seen, with the top 15 PFAS having functional groups such as amide, ketone, esther, and ether. Additionally, Log*p* values have been used to assess bioaccumulation potential, with values exceeding 5 often correlating with prolonged persistence and bioaccumulation [57,58]. The top-binding PFAS for all receptors displayed Log*p* values greater than 5, emphasizing their substantial risk of bioaccumulation. For example, compounds such as 662-28-2 and 306-91-2 showed the strong bioaccumulation potential of the top PFAS CAR binders, while compounds like 446043-85-2 and 347838-21-5 illustrated the same for the top PFAS PXR binders.

#### 2.5.2. Hydrophobicity Analysis of Other EDC Classes

An examination of the predicted Log*p* values for the top-binding plastic chemicals reveals a consistent trend similar to the trend observed with PFAS. Characterized by high Log*p* values, the top binders among the plastic chemicals were consistently hydrophobic, similar to what was observed for the top PFAS binders. Analysis of the predicted Log*p* values across different classes of EDCs supports a universal hydrophobic profile for the top binders. For example, the top 15 PBDEs had an average Log*p* value of 7.3, reinforcing the trend of high hydrophobicity as a common feature among the strongest binders across diverse EDC classes. This universal hydrophobic profile suggests that hydrophobic interactions play an important role in the binding interactions of various EDCs with nuclear receptors such as CAR, and influence their biological activity and persistence in the environment. In addition to the common hydrophobic characteristics and interactions that are observed across EDCs, there is also a notable structural similarity among the strongest binders within each class. Specifically, a cyclic structural property is prevalent among the top binders across various classes of EDCs. The strongest PFAS, PBDE, and phthalate had similar cyclic features. These cyclic structures play a significant role in hydrophobic interactions, which are necessary for the stronger binding affinity of these compounds.

### 2.6. Functional Group Analysis

#### 2.6.1. Functional Group Analysis of PFAS

The PFAS with the highest binding affinities to CAR were uniformly hydrophobic, lacking any detectable functional groups and consisting solely of carbon and fluorine atoms in cyclic rings. This is consistent with the hydrophobic nature of CAR’s binding pocket, where 71% of the amino acid residues are classified as hydrophobic. In contrast, for the PXR, only 3 of the top 15 PFAS were similarly hydrophobic and devoid of functional groups, while the remaining 12 compounds contained polar functional groups such as alcohols or amides. Similarly, the majority of the top 15 PFAS bound to the AR were exclusively hydrophobic, composed of carbon and fluorine atoms. Among these top binders, only three compounds contained functional groups, specifically alkene, sulfide, and ether groups. A similar trend was observed for PPARδ, where three of the top five strongest-binding PFAS were composed solely of carbon and fluorine atoms. However, deviations from this pattern were seen with PPARα and PPARγ, where the top 15 PFAS included a broader range of functional groups containing sulfur, oxygen, and nitrogen atoms. This divergence highlights the variability in functional group contributions to binding affinity across different nuclear receptors, particularly PPARα and PPARγ, compared to CAR, AR, and PPARδ, where hydrophobicity and the absence of polar functional groups dominate binding interactions. The binding affinities shown correspond to PFAS compounds containing the listed functional group and represent their average affinity for the seven studied receptors (Table 3). The data points for the functional group analysis can be found in Supplementary Table S2. The 2D structures for the top 15 PFAS bound to each receptor can be found in Supplementary Figures S17–S23.

The average docking scores for various functional groups across different nuclear receptors were analyzed. For CAR, sulfonic acid had the most negative average docking score at −9.06 kcal/mol, indicating a strong interaction. Sulfonic acid is a functional group found in certain widely studied PFAS including PFOS. Anhydrides and sulfonamides also demonstrated strong binding to CAR, with average docking scores of −8.81 kcal/mol and −8.68 kcal/mol, respectively. In contrast, thioketone and thiol displayed the weakest binding affinities, with scores of −6.16 kcal/mol and −6.50 kcal/mol, respectively. For PPARγ, carbamate was found to have the strongest average docking score of −9.00 kcal/mol, followed closely by urea and anhydride, both with an average score of −8.50 kcal/mol. Similarly, in RORγt, carbamate and sulfonic acid had the most negative docking scores at −9.00 kcal/mol. Urea and sulfonamide followed closely, each with an average score of −8.90 kcal/mol. For PXR, urea-containing compounds demonstrated the strongest binding strength, showing urea’s consistent role in driving strong receptor–ligand interactions across multiple nuclear receptors. Specific functional groups, particularly sulfonic acid and urea, are important in mediating strong binding affinities to key nuclear receptors involved in PFAS-related toxicity. Similarly, carbamate was identified as the functional group with average highest binding affinity score of −8.806 kcal/mol for PFAS docked to PPARα. Urea and sulfonic acid proceeded with scoring high binding affinities of −8.761 and −8.577 kcal/mol, respectively. Sulfonic acid, urea, and carbamate demonstrated strong binding affinities to CAR, PPARγ, RORγt, and PXR, showing their importance in PFAS-related toxicity.

#### 2.6.2. Functional Group Analysis of Plastic Compounds

The average docking scores of nanoplastic-related compounds, categorized by functional groups, were analyzed across various nuclear receptors. For CAR, phenyl-containing compounds displayed high binding affinity, with an average docking score of −7.6 kcal/mol across 763 compounds, suggesting that hydrophobic and aromatic interactions play a significant role in stabilizing these compounds in CAR’s binding pocket. Alkyne-containing compounds also demonstrated strong binding to CAR, with the most negative average docking score of −7.71 kcal/mol, though this result was derived from a smaller sample size of seven compounds. For PPARγ, sulfoxide and thioketone had the most negative docking scores, both at −8.3 kcal/mol, indicating strong binding affinity. Phenyl-containing compounds followed with an average docking score of −7.5 kcal/mol. Similarly, RORγt displayed the strongest binding interactions with sulfoxides, which had an average docking score of −8.3 kcal/mol. Alkyne-containing compounds followed closely with an average score of −8.1 kcal/mol, demonstrating the significance of both sulfur-based and hydrophobic groups in binding to these receptors. For PPARα, sulfonamide- and sulfonic acid-containing compounds had the strongest binding affinities, with docking scores of −7.35 kcal/mol and −7.29 kcal/mol, respectively. A similar trend was observed for PPARδ, where sulfoxides and thioketones demonstrated the strongest average docking scores, consistent with PPARγ. Additionally, for PXR, sulfonamide-, sulfone-, and sulfonic acid-containing compounds showed the greatest binding affinities, revealing a common trend of sulfur-based functional groups contributing to strong receptor–ligand binding. In contrast, thiol-containing compounds consistently have the weakest binding affinities across all nuclear receptors, with the lowest average docking score of −5.09 kcal/mol for CAR. This suggests that thiol groups do not form strong interactions with these receptors, which may be partially due to the small sample size of 16 thiol-containing compounds. This trend of weak binding for thiol-containing compounds was observed across all receptors analyzed.

### 2.7. Prevalence Analysis of Top Binders Across NRs

We created heat maps for all EDC classes using their respective binding affinities. For nanoplastics, PFAS, and PBDE compounds, the top 25 were analyzed to assess the frequency of ligands as top binders across the studied NRs. Heat maps were created for the top 50 binders for every NRs. This was used to investigate compounds that appeared as top binders for multiple EDC classes. For PFAS, no compound appeared more than three times among the EDC classes (Table 4). Compound 662-28-2, the top binder for CAR, was the 18th strongest binder for PXR and the 16th strongest binder for AR. Compound 548470-06-0, the top binder for PPARα, was also the third strongest binder for AR and the eighth strongest binder for PPARδ. These results show the ability of certain PFAS to bind strongly to multiple NRs. For PBDEs, one compound was present as a top binder for five nuclear receptors. 366791-32-4, the top binder for PPARδ, was also the 6th strongest binder for PXR, 10th for PPARα, 19th for PPARγ, and the 3rd strongest binder for ROR, demonstrating the strong binding potential of 366791-32-4 across the spectrum of compounds (Table 5). For plastic chemicals, perfluorotetradecanoic acid was present as a top binder for all nuclear receptors. 376-06-7, a PFAS, was present in all NRs, showing the ability of certain PFAS to strongly bind to multiple NRs. Furthermore, compound CAS 1533-45-5 was present in four NRs: PPARα, PPARγ, PPARδ, and RORγt (Table 6). These results demonstrate the ability of certain compounds to bind strongly to multiple NRs, showing their strong competitive binding potential, risk of overactivation, and carcinogenic potential.

For PFAS pesticides, a trend among ROR, PXR, and the PPARs was observed. Novaluron, Noviflumuron, and Oxathiapiprolin were all observed to demonstrate strong binding potential to these NRs while demonstrating relatively weak binding potential to AR and CAR (Table 7). Prodiamine demonstrated weak binding strength for all NRs. The heat map for BPAs revealed 2,2-bis(2-hydroxy-5-biphenylyl)propane as having strong binding potential for all nuclear receptors but AR (Table 8). No other clear trends were observed for the BPAs. The heat map analysis conducted on the phthalates reveals similar trends among docking scores for all receptors. We found that dicyclohexyl phthalate was revealed to be the top binder for all receptors, showing the strong binding potential of this phthalate for all NRs (Table 9). Similarly, butyl benzyl phthalate was found to be a strong binder for all NRs as well. A trend among weak binders was also observed for the phthalates, as diethyl phthalate and Bis(2-methoxyethyl) phthalate were observed to be weak binders for all NRs.

### 2.8. Experimental Analysis of NR Activation and NR Transcription Pathway Analysis of Phthalates

#### 2.8.1. Experimental Analysis of Phthalate Binding Activity to NRs

**Figure 8:**
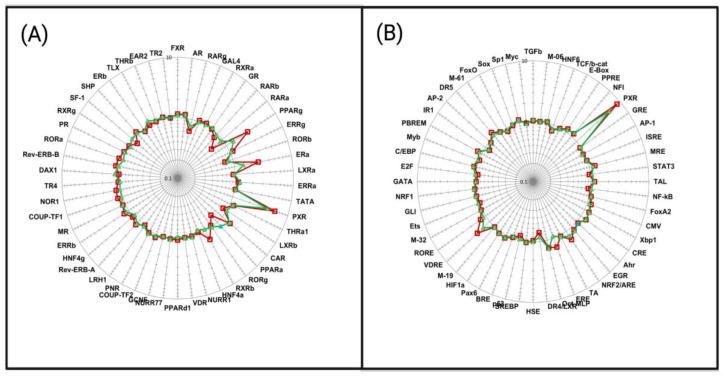
Effect of DCHP and DEHP (10 mM, 16 h) on NR activity in HepG2 cells. HepG2 cells were transiently transfected with the optimized trans-FACTORIALTM library. After twenty four hours, the transfection cells were washed, supplied with fresh low-serum (1% FBS, charcoal-stripped) culture medium, and treated with an inducer for 16 h. The profile of the trans-FACTORIALTM activities was determined as a fold of induction values versus vehicle-treated (DMSO) control cells. The graph shows fold-induction data plotted in logarithmic scale. (**A**) Red-colored data represent dicyclohexyl phthalate to 50 human NRs. Green-colored data represent bis-(2-ethylhexyl) phthalate to 50 human NRs. (**B**) Red-colored data represent various transcription factors in response to dicyclohexyl phthalate in relation to 50 human NRs. Green-colored data represent various transcription factors in response to bis-(2-ethylhexyl) phthalate in relation to 50 human NRs.

Our in silico docking analysis of seven NRs revealed a high binding affinity for dicyclohexyl phthalate (DCHP) (Figure 3). DCHP was identified as the top-binding phthalate across all receptors. Additionally, bis-(2-ethylhexyl) phthalate (DEHP) is known to bioaccumulate, a factor that cannot be accounted for in molecular simulations [59]. Due to their bioaccumulation and strong binding capabilities to NRs relative to other EDC classes, DCHP and DEHP were candidates for an in vitro NR activation assay. Inspired by these results, we performed in vitro assays to assess the impact of DEHP, a known endocrine disruptor, and dicyclohexyl phthalate, a strong binder to all studied NRs [60]. These assays tested the activation of both phthalates on 50 NRs, focusing on NR activation and transcription-pathway responses. The assay yielded PXR having the strongest activation when exposed to DCHP and DEHP. Previous studies have also identified dicyclohexyl phthalate as a PXR-selective agonist in wild-type mice [61]. To further correlate our experimental results, we performed molecular docking of DCHP and DEHP to the following NRs: RARγ and RARβ, RORβ, and RXRβ, SF-1, and ERα. Consistent with the experimental results in Figure 8, the aforementioned receptors showed greater affinity for DCHP than DEHP (Table 9).

DCHP displayed both agonistic and antagonistic effects on NRs. Specifically, for RXRβ and ERα, DCHP acted as an agonist (Figure 8). DCHP showed relatively high docking scores when bound to the LBDs of RXRβ and ERα. Given the phthalate’s high bioaccumulation, its exposure to these NRs suggests a strong binding potential. Additionally, DCHP showed a high binding affinity of −9.9 kcal/mol with RARβ and presented inhibitory effects (Table 9). Similarly, DCHP binds with high affinity to RARγ, RORβ, and SF-1, with high docking scores, and displayed antagonistic effects (Table 9). Further, the assay demonstrated various effects of DCHP binding on the seven NRs studied. DCHP showed strong agonistic effects on PXR and PPARγ. Similarly, CAR and AR showed a noticeable agonistic effect, with strong docking scores. PPARδ displayed neither an agonistic nor antagonistic effect with DCHP. RORγ and PPARα demonstrated a slight antagonistic activity, with DCHP interacting with high affinities from our docking results. Thus, the assay, in combination with in silico data, shows that DCHP can cause a variety of activation effects on hNRs. The agonist effects of DEHP and dicyclohexyl phthalate on both PPARγ and CAR raise significant toxicity concerns due to the association between receptor activation and hepatotoxicity [61,62]. Although having less functional activity, DEHP also showed both agonistic and antagonistic effects on NRs. DEHP displayed strong agonistic effects on PXR, corresponding to a docking score of −8.6 kcal/mol. Further, CAR and AR showed agonistic effects with moderate binding affinities. ERα demonstrated relatively low agonistic activity when binding with DEHP (Figure 8B). In contrast, DEHP interaction with SF-1 indicated no agonistic or antagonistic response. Similarly, PPARδ and RORγ displayed a neutral effect with DEHP. Receptors RARγ, RARβ, RORβ, and RXRβ all experienced inhibition upon DEHP binding to the LBD, with RARγ and RORβ suggesting stronger antagonistic effects. Therefore, the data illustrate that DEHP can cause a variety of activation effects on NRs.

The HepG2 cells used in the assay play a major role in assessing NR activation by DCHP and DEHP. HepG2 cells express a variety of the studied NRs, including PXR, CAR, PPARα, and RARβ [63,64]. PXR is localized in the nucleus of monolayer-cultured HepG2 cells and has been found to be expressed in liver cells, the intestine, and fetal tissue [65,66]. PXR was shown to have a strong agonistic response to both DCHP and DEHP, indicating that these phthalates have the strong ability to disrupt liver, intestine function, and fetal development [65]. Similarly, CAR also showed moderated agonistic effects to DCHP and DEHP. Due to CAR’s expression in the brain, heart, lung, liver, testis, pancreas, and kidney, the studied phthalates have the potential to disrupt such organs [67,68]. Additionally, PPARα is expressed in the liver, heart, skeletal muscle, brown adipose tissue, and kidney [69]. DCHP and DEHP showed agonist and antagonist activity towards PPARα, respectively (Table 10). This illustrates these two phthalates’ strong ability to activate or inhibit biological activity in muscles and major organs. Nuclear receptor HN4α, which was studied in the HepG2 assay, has been shown to play a major role in the expression of the retinoic acid receptor β (RARβ) gene in hepatocytes [70]. The RAR receptor family is highly expressed in various tissues including the cerebrum and eye [71]. Notably, psychiatric conditions such as schizophrenia, autism, alcoholism, bipolar disorder, and attention deficit hyperactivity disorder (ADHD) have been linked to malfunction of the RA/RAR signaling axis [71]. Both studied phthalates showed antagonist activity towards RARβ, showing the ability of these phthalates to potentially affect neural function and explaining its correlation to psychiatric disorders.

#### 2.8.2. Experimental Analysis of Phthalate Modulation of Transcription Factor Pathways

The transcription factor pathway assay assessed the gene expression regulation of transcription factor pathway modulation of 52 different transcription factors in HepG2 cells. Exposure to DEHP and DCHP showed a positive fold induction of VDRE and PXR (Figure 8B). When bound and activated by DEHP and DCHP, PXR translocates to the nucleus and binds to DNA response elements as a heterodimer with Retinoid X Receptor (RXR) [72]. PXR then recruits several coactivators, including steroid receptor coactivator 1 (SRC-1), TIF2/GRIP1 (SRC-2), and peroxisome proliferator-activated receptor gamma coactivator 1 alpha (PGC-1α) [72]. These coactivators regulate zinc finger motifs and xenobiotic response elements (XREs) on the promoters of PXR target genes [72]. This pathway emphasizes the role of PXR and its coactivators in controlling the cellular response related to xenobiotic metabolism and detoxification. Therefore, if highly accumulating phthalates can activate these pathways, NR activation may lead to unwanted downstream effects. Similarly, DCHP and DEHP activate VDR, forming heterodimers with RXR and attaching to VDREs. When VDR is bound to DNA, it acts as a molecular activator for nuclear signaling of 1α,25(OH)2D3 (calcitriol), which determines its activation state for chromatin regions containing calcitriol target genes [73]. This VDR-mediated pathway demonstrates how exposure to DCHP and DEHP may influence the transcriptional regulation of calcitriol target genes, which affects cellular processes dependent on vitamin D signaling. Similarly, Issa et al. discussed salpn-induced VDRE-mediated pathways through a CPTM (chemo-phenotypic-based toxicity model), finding that non-endogenous ligands, such as phthalates and salpn, can activate the NR pathway [38]. Thus, this study showed how NR activation by EDCs such as DCHP and DEHP could disrupt VDR’s role in gene expression. As shown by these two major pathways, DEHP and DCHP have the potential to alter the long-term regulation of NR-related gene expression, which may lead to carcinogenic consequences.

## 3. Materials and Methods

### 3.1. Receptor and Endogenous Ligand Preparation

The nuclear receptor structures were retrieved from the Protein Data Bank (PDB) [74]. All non-standard amino acid residues were removed from the receptor file, including extra side chains, water molecules, and hetero/homodimers using ChimeraX 1.8, 2024. To prepare the receptors for molecular docking simulations, Autodock 4.2 Tools were employed [75]. The hydrogens were added to the receptor structures, and Kollman charges were assigned [76]. The protein structures were then exported in .pdbqt format. The ligand-binding domain for each receptor was determined, and the grid box was recorded. The specific grid box dimensions and center coordinates for each receptor docking are listed in Supplementary Table S1.

### 3.2. Preparation of PFAS

A total of 14,735 PFAS were obtained from the Environmental Protection Agency (EPA) CompTox dashboard PFAS structure list as an Excel file [77]. PFAS without valid SMILES notation were discarded from the data. Using Python code (Supplementary Code S1), 14,591 PFAS were successfully converted from the SMILES notation to the PDB format. The compounds were then converted from .pdb format to .pdbqt format and energy-minimized using OpenBabel (Version 2.3.3) using Python code (Supplementary Code S2) [78]. During the energy minimization and conversion process, PFAS were minimized using the general amber force field (GAFF) [79]. The structure of the PFAS prepared can be found in Supplementary Code S1.

### 3.3. PFAS Structural Analysis

Physicochemical characteristics of PFAS were obtained using Python code (Supplementary Code S3). Using the SMILES notation, the polar surface area (PSA) and the log of the partition coefficient of a solute between octanol and water (Log*p*) were predicted to quantify the polarity of PFAS to understand the impact of polarity and hydrophobicity on the binding affinity of EDCs to NRs. The functional groups present in PFAS were also determined to investigate the impact that they have on the binding potential of PFAS to NRs using Python code (Supplementary Table S2 and Code S4). The following functional groups were considered: alcohol, aldehyde, ketone, carboxylic acid, amine, ether, ester, amide, alkene, alkyne, thiol, sulfoxide, sulfone, nitrile, nitro, phenyl, halide, anhydride, isocyanate, urea, and carbamate.

### 3.4. Preparation of Plastic Chemicals

The plastic chemicals were retrieved in SMILES format provided in the Plastic Chemicals Database, as documented by Wagner et al. [80]. For further analysis, only plastic chemicals with a molecular weight of 175 to 1000 g/mol were selected for pdbqt preparation. From the database, a total of 1927 plastic chemicals met this criterion and were subsequently converted for further analysis. The structure of plastic chemicals prepared can be found in Supplementary Code S1.

### 3.5. Preparation of Other EDC Classes

To retrieve the pdb formats for phthalates, bisphenols, PBDEs, and PFAS pesticides, web scraping techniques were implemented. Python code in Supplementary Code S5 and S6 used the PubChem Database to obtain the SMILES notation and molecular weight for each ligand, respectively [81]. Using Python code (Supplementary Code S1), the EDCs were successfully converted from the SMILES notation to the PDB format. The compounds were then converted from pdb to pdbqt and energy-minimized using OpenBabel (Version 2.3.3) through Python code (Supplementary Code S2). A total of 209 PBDEs, 58 bisphenols, 38 PFAS pesticides, and 6 phthalates were analyzed. The structure of the EDC classes prepared can be found in Supplementary Code S1.

### 3.6. Quantification of Amino Acid Residue Interactions

To identify the key amino acid residues that interact with PFAS and other EDCs, the top 15 binders for each toxicant class were downloaded using Python code (Supplementary Code S7). The frequency of each amino acid interaction was calculated using Python code (Supplementary Code S8) through Chimera 1.7.1 automation for the top 15 binders [82]. The nuclear receptor residues interacting within 5 Å units of the ligand were selected and output to PDB format. Python code (Supplementary Code S9) was implemented to convert the interacting residues into FASTA format.

### 3.7. Classification of Binding Strength to NRs

The docking results of all EDC groups were compared to both endogenous and synthetic ligands. For the classification, docking scores were used as a quantitative measure of binding affinity. Specifically, chemicals that showed docking scores exceeding the mean docking score of the reference ligands, comprising both endogenous and synthetic ligands, were categorized as strong binders. Conversely, chemicals with docking scores within a margin of 10% below the average docking score of the reference ligands were classified as moderate binders. Finally, those chemicals with docking scores falling more than 10% below the average docking score of the reference ligands were considered weak binders. This stratification, as performed by Azhagiya Singam et al., provides a clear framework for assessing the relative binding affinities of various EDCs in relation to standard ligands [37].

### 3.8. Molecular Docking Using Autodock Vina

Using Python code (Supplementary Code S10), molecular docking simulations were performed using Autodock Vina to study the interactions between NRs and EDCs. The simulations were focused on the orthosteric sites of the receptors. Grid box coordinates are defined in Supplementary Table S1. The docking protocol and Vina predictive accuracy were validated through cross-docking experiments with known native ligands for the NRs. The reference ligands demonstrated binding conformations within 2.5 Å of the co-crystal structures, validating the accuracy of molecular docking simulations in predicting protein–ligand interactions and binding affinity. The complete results of AutoDock Vina are compiled and displayed in Supplementary Tables S3–S8.

### 3.9. Carbon Chain Length Determination

Python code (Supplementary Code S11) was used to calculate the longest fluorinated chain from the SMILES format of PFAS. The code identifies the longest continuous carbon chain by iterating through neighboring carbon atoms that have at least one attached fluorine. The carbon chain length for each PFAS was matched to its binding affinity (Supplementary Table S9).

### 3.10. Human Nuclear Receptors and Transcription Factor Activity Assays

We used Attagene Inc., Morrisville, NC, USA, FACTORIAL TF and NR assays (cis-FACTORIAL™ and trans-FACTORIAL™) to assess how a compound influences cellular transcription factor (TF) and NR activities. The assays provide a reporter detection platform that allows for the simultaneous measurement of multiple reporter signals in a single cell sample. This ensures consistent detection accuracy across multiple targets. Detailed descriptions of the FACTORIAL TF and NR assays can be found in previous publications [83,84,85,86,87]. To evaluate the effect of DEHP and DCHP (10 mM, 16 h) on NR activity in HepG2 cells, we used the trans-FACTORIAL™ assay. HepG2 cells were transiently transfected with an optimized library of NR reporters. After 24 h, the cells were washed, placed in a fresh low-serum medium (1% FBS, charcoal-stripped), and treated with the compound for 16 h. NR activity was measured as the fold induction relative to vehicle-treated (DMSO) control cells. Similarly, we used the cis-FACTORIAL™ assay to assess the effect of DEHP and DCHP on TF activity in HepG2 cells. The cells were transfected with an optimized TF library, washed after 24 h, and treated under the same conditions as above. TF activity was also measured as the fold induction relative to DMSO controls.

## 4. Conclusions

In summary, this study provides new insights into the interactions between EDCs and the studied NRs. Through our validated molecular docking, which involved investigating the protein–ligand binding interactions between 16,829 EDCs and 7 NRs, we observed that PFAS showed strong binding interactions with PXR, CAR, PPARα, PPARδ, PPARγ, and RORγt, while having weaker interactions with AR. Our findings emphasize the potential of EDCs to competitively bind to NRs, resulting in potential endocrine disruption. Additionally, fluorine atom count, fluorinated carbon chain length, and hydrophobicity were analyzed to understand PFAS binding strength. Functional group analysis revealed the importance of sulfur- and nitrogen-based functional groups in the binding strength of EDCs. PFAS, in particular, showed stronger binding to receptors with larger ligand-binding domains, such as PXR and PPARs, when having longer fluorinated carbon chains. The unique electronegativity of fluorine atoms allows for stronger hydrogen bonds with polar residues, improving the binding stability and bioaccumulation potential of these compounds. This preference for long-chain, fluorinated structures suggests that chemical modifications in PFAS might greatly impact their environmental persistence and bioaccumulation in organisms. PXR and PPARγ, which have large binding domains, had higher binding affinities for a range of EDC classes, whereas AR exhibited weaker binding interactions, potentially due to its smaller ligand-binding domain. Due to structural differences in LBDs, which affect structural compatibility and receptor specificity, EDCs induce different downstream effects. Hydrophobic interactions also played a major role in determining the binding affinities of EDCs to NRs. The strong correlation between specific structural features, such as polycyclic rings and extended fluorinated carbon chains, shows the importance of these properties in relation to binding affinity. Further analysis revealed that amino acid residues like leucine, phenylalanine, and isoleucine within the ligand-binding domains of the receptors play a key role in these interactions. The prevalence of these hydrophobic residues suggests that EDCs with similar characteristics are more likely to strongly bind and activate these receptors, as indicated by greater Log*p* values for all studied NRs.

The investigation of 7 distinct NRs and the potential interactions with 16,829 EDCs highlights a unique strength of our study. The diversity of the EDC classes is novel, as previous studies have focused on singular classes of compounds [37,65]. The wide binding spectrum of EDCs was an additional strength, which allowed us to factor in specific binding interactions. Local binding site flexibility was accounted for in this study; however, future work should incorporate the full flexibility of top EDC-binding complexes, which could be achieved by incorporating MM-GBSA or MM-PBSA simulations. Though we established NR and transcription factor assays to test top phthalate chemicals, it requires a specific binding assay. Further, to validate these computational findings, additional in vitro binding studies should target PFAS and plastic-associated chemicals, with specific attention to the chemicals that bind strongly to multiple NRs. Testing allosteric binding sites is also important, as they may serve as secondary activation domains, with EDC binding to both sites possibly leading to NR activation. Allosteric sites have been studied for NRs like VDR, PPARγ, and PXR, but only with PFAS [45]. Future research should explore allosteric sites in other NRs as well as examine the binding of different EDC classes at these sites.

These results emphasize the cumulative effects of EDC exposure, especially in cases of co-exposure to multiple EDC classes. This indicates the need for further in vitro studies and regulatory measures to control the risks posed by these EDCs. The results from this study suggest that future research should prioritize exploring the toxicological profiles of EDCs, considering factors like bioaccumulation potential, receptor-specific activation patterns, and possible carcinogenicity. Certain PFAS and plastic chemicals showed high binding affinities across multiple NRs, indicating that cumulative exposure to these substances could disrupt various cellular signaling pathways simultaneously. The implications of these findings are important, as the interaction of these EDCs with multiple receptors could worsen their toxicological effects, justifying further investigation into the cumulative health risks posed by prolonged exposure to such chemicals. Further, dicyclohexyl phthalate was identified as the top phthalate binder for all NRs investigated. PFTA was a plastic chemical predicted to be a top binder for all NRs. Similarly, compound 366791-32-4 of the PBDE class was present as a top predicted binder for five NRs. Dicyclohexyl phthalate and DEHP have strong agonistic effects on certain nuclear receptors, mainly PXR and PPARγ. Supported by in vitro assays, DEHP and dicyclohexyl phthalate produced strong activation of PXR and PPARγ, while showing slight activation of CAR and AR. Dicyclohexyl phthalate showed antagonistic effects on PPARα, contrasting with the slight activation of PPARα by DEHP. The activation of key NRs such as PXR and PPARγ by DEHP and DCHP presents significant endocrine-disrupting concerns. The endocrine-disrupting potential of DCHP and DEHP is further evidenced by DCHP and DEHP resulting in high levels of fold induction of the PXR TFs. Overall, it is necessary to further examine the mechanisms by which these EDCs can activate major hNRs.

## Figures and Tables

**Table 1:** Percentage of NRs with strong, moderate, and weak PFAS binders with count for PXR, CAR, PPARα, PPARγ, PPARδ, AR, and RORγt.

Receptor	Strong Binder (%)	Moderate Binder (%)	Weak Binder (%)
PXR	13.1	16.8	70.2
CAR	7.8	13.9	78.3
PPARα	5.8	11.4	82.7
PPARγ	16.9	19.0	64.1
PPARδ	8.7	12.0	79.3
AR	0.5	2.1	97.4
RORγt	12.8	13.5	73.7

**Table 2:** Percentage of NRs with strong, moderate, and weak nanoplastic binders with count for PXR, CAR, PPARα, PPARγ, PPARδ, AR, RORγt.

Receptor	Strong Binder (%)	Moderate Binder (%)	Weak Binder (%)
PXR	6.5	10.6	82.8
CAR	1.5	4.2	94.3
PPARα	0.9	3.8	95.3
PPARγ	8.2	9.9	81.8
PPARδ	2.6	7.1	90.3
AR	0.1	0.4	99.5
RORγt	2.1	6.2	91.7

**Table 3:** Average docking score (kcal/mol) of functional groups for seven studied NRs.

Functional Group Present in PFAS	CAR	PXR	PPARα	PPARγ	PPARδ	AR
Alcohol	−8.17	−7.9	−7.79	−7.67	−7.96	−7.78
Aldehyde	−7.46	−7.24	−7.11	−7.09	−7.29	−7.32
Alkene	−7.82	−7.49	−7.28	−7.34	−7.38	−7.52
Alkyne	−7.18	−7.03	−6.72	−7.05	−6.95	−7.00
Amide	−8.27	−8.61	−8.32	−8.38	−8.59	−7.99
Amine	−7.78	−7.66	−7.48	−7.47	−7.56	−7.65
Anhydride	−8.82	−8.58	−8.2	−8.47	−8.49	−8.23
Carbamate	−8.67	−8.93	−8.81	−8.96	−9.22	−7.88
Carboxylic Acid	−8.31	−7.95	−7.86	−7.66	−8.03	−7.91
Disulfide	−7.09	−6.85	−6.36	−6.44	−6.50	−6.77
Ester	−8.06	−7.79	−7.76	−7.9	−7.87	−7.71
Ether	−7.89	−7.63	−7.51	−7.64	−7.62	−7.56
Halide	−7.82	−7.57	−7.36	−7.39	−7.49	−7.52
Isocyanate	−7.29	−6.98	−6.87	−6.971	−6.92	−7.32
Ketone	−8.11	−7.9	−7.78	−7.81	−7.92	−7.8
Nitrile	−7.06	−7.11	−6.84	−6.8	−6.90	−6.97
Phenyl	−8.34	−8.28	−7.86	−8.04	−8.15	−7.82
Sulfide	−7.59	−7.46	−7.28	−7.15	−7.40	−7.3
Sulfinate Ester	−6.54	−6.19	−6.29	−6.52	−6.12	−6.59
Sulfonamide	−8.68	−8.49	−8.38	−8.44	−8.72	−8.04
Sulfonate Ester	−8.26	−7.96	−7.65	−8.13	−7.92	−7.98
Sulfone	−8.67	−8.37	−8.2	−8.26	−8.50	−8.11
Sulfonic Acid	−9.06	−8.6	−8.58	−8.41	−8.85	−8.45
Sulfoxide	−7.9	−7.77	−7.58	−7.41	−7.89	−7.63
Thioketone	−6.16	−6.11	−5.84	−6.06	−5.80	−6.11
Thiol	−6.5	−6.37	−6.12	−6.18	−6.14	−6.44
Urea	−7.54	−9.28	−8.76	−8.47	−8.94	−7.36

**Table 4:** Heat map of the top 25 binders of PFAS bound to CAR, PXR, AR, PPARα, PPARγ, PPARδ, and RORγt. Light yellow represents compounds that appear twice between the NRs. Light orange represents compounds that appear three times.

	CAR	PXR	AR	PPARα	PPARγ	PPARδ	RORγt
1	662-28-2	5366-99-4	306-91-2	548470-06-0	NOCAS_1026885	21674-38-4	862130-96-9
2	306-91-2	61778-79-8	86630-50-4	547748-28-7	57589-85-2	57101-59-4	86630-50-4
3	307-08-4	548470-06-0	558-64-5	21674-38-4	151707-04-9	347838-21-5	88951-08-0
4	118914-94-6	NOCAS_871520	119107-96-9	NOCAS_1027003	749924-57-0	25761-65-3	NOCAS_1026885
5	558-64-5	347838-21-5	61855-74-1	1075687-38-5	919293-76-8	230295-10-0	2663-96-9
6	118914-93-5	NOCAS_1026885	306-99-0	151707-03-8	1075687-38-5	862589-19-3	305849-27-8
7	97571-69-2	131662-80-1	306-95-6	25761-65-3	646533-88-2	23790-50-3	15038-90-1
8	33021-47-5	146304-71-4	NOCAS_1027599	61798-68-3	919293-77-9	548470-06-0	3824-74-6
9	306-99-0	126565-13-7	NOCAS_1026765	464-34-6	919293-78-0	NOCAS_1027003	61855-74-1
10	118945-64-5	446043-85-2	95827-25-1	NOCAS_1035154	55987-23-0	1127427-75-1	819792-77-3
11	307-07-3	NOCAS_1026915	118914-93-5	NOCAS_1026885	231953-37-0	146304-71-4	862130-87-8
12	105462-77-9	NOCAS_1026998	119141-86-5	55987-23-0	68541-01-5	649561-66-0	862130-95-8
13	125061-94-1	125061-94-1	114832-09-6	160248-96-4	151707-03-8	548434-15-7	862133-29-7
14	61855-74-1	87667-00-3	119141-87-6	NOCAS_1035147	675583-26-3	126565-13-7	151707-04-9
15	119107-96-9	61547-75-9	306-92-3	548455-52-3	667938-69-4	646533-88-2	61547-75-9
16	118945-65-6	NOCAS_1027048	662-28-2	916770-15-5	134880-89-0	231630-88-9	862130-67-4
17	95827-25-1	60131-74-0	60433-12-7	4314-47-0	61547-75-9	677033-16-8	NOCAS_1027003
18	NOCAS_1027599	662-28-2	306-94-5	1555-24-4	74582-85-7	61778-79-8	112242-89-4
19	444658-67-7	NOCAS_1026766	60433-11-6	548478-86-0	915-76-4	548478-86-0	24768-65-8
20	17051-14-8	876517-48-5	86714-34-3	305849-27-8	200862-70-0	1127427-64-8	146304-71-4
21	119141-86-5	919489-99-9	444658-67-7	57338-64-4	1555-24-4	61798-68-3	55987-23-0
22	306-95-6	116265-66-8	107349-88-2	NOCAS_1035142	234449-42-4	848352-66-9	862133-15-1
23	119107-97-0	48240-25-1	118945-64-5	NOCAS_1035206	864149-62-2	548455-85-2	NOCAS_1026997
24	114832-09-6	304658-31-9	49771-67-7	NOCAS_1035822	881689-01-6	NOCAS_1026885	306-99-0
25	68697-63-2	NOCAS_1009912	105462-77-9	240121-57-7	132877-69-1	749924-57-0	4314-47-0

**Table 5:** Heat map of the top 25 binders of PBDE bound to CAR, PXR, AR, PPARα, PPARγ, PPARδ, and RORγt. Light yellow represents compounds that appear twice between the NRs. Light orange represents compounds that appear three times. The dark orange represents compounds that appear 4 times. The darkest orange represents compounds appearing 5 times.

	CAR	PXR	AR	PPARα	PPARγ	PPARδ	RORγt
1	446255-01-2	446255-18-1	147217-74-1	405237-85-6	446254-32-6	366791-32-4	446255-08-9
2	446254-72-4	446254-80-4	446254-24-6	446254-95-1	68631-49-2	373594-78-6	373594-78-6
3	218304-36-0	93703-48-1	337513-55-0	51452-87-0	337513-67-4	446254-48-4	366791-32-4
4	446254-55-3	446254-86-0	337513-68-5	446254-71-3	171977-44-9	405237-85-6	446254-82-6
5	446254-63-3	373594-78-6	446254-62-2	83694-71-7	446254-80-4	337513-68-5	446254-31-5
6	117948-63-7	366791-32-4	446254-45-1	147217-72-9	189084-59-1	446254-82-6	446255-18-1
7	446254-33-7	446255-07-8	446254-28-0	446254-70-2	46438-88-4	103173-66-6	446254-27-9
8	446254-17-7	446254-45-1	147217-76-3	446255-17-0	60348-60-9	446254-77-9	446254-70-2
9	446254-30-4	446254-28-0	446254-19-9	446254-97-3	147217-77-4	446254-84-8	446254-71-3
10	327185-09-1	189084-61-5	446254-15-5	366791-32-4	337513-54-9	446254-32-6	182677-28-7
11	446254-21-3	446254-29-1	49690-94-0	446254-27-9	446254-65-5	446254-39-3	446254-48-4
12	446254-32-6	446254-31-5	189084-62-6	446254-38-2	5436-43-1	446254-31-5	405237-85-6
13	446254-35-9	446254-39-3	147217-75-2	446254-48-4	189084-61-5	446254-64-4	446254-80-4
14	446254-36-0	446254-82-6	407606-55-7	407606-55-7	337513-55-0	446254-18-8	93703-48-1
15	446254-37-1	446254-64-4	337513-75-4	147217-73-0	101-55-3	189084-59-1	446254-28-0
16	446254-68-8	446254-50-8	446254-30-4	446255-10-3	93703-48-1	147217-77-4	446254-81-5
17	446254-95-1	446255-13-6	337513-53-8	446255-18-1	337513-72-1	446255-13-6	327185-11-5
18	446255-00-1	446254-19-9	446254-25-7	446254-45-1	446254-70-2	446254-20-2	446254-38-2
19	446255-12-5	147217-81-0	337513-56-1	446254-39-3	366791-32-4	446254-51-9	446254-83-7
20	446255-20-5	446254-27-9	446254-18-8	446254-50-8	407606-55-7	327185-11-5	446254-34-8
21	337513-66-3	446254-38-2	337513-82-3	446254-53-1	446254-39-3	60348-60-9	446254-78-0
22	51930-04-02	446254-71-3	147217-81-0	446254-32-6	259087-35-9	446254-38-2	446255-09-0
23	189084-60-4	446254-42-8	446255-04-5	147217-71-8	417727-71-0	446255-18-1	446254-96-2
24	337513-68-5	446254-48-4	446254-27-9	101-55-3	147217-75-2	147217-76-3	147217-76-3
25	446254-22-4	446255-08-9	446254-53-1	446254-84-8	337513-66-3	446254-86-0	147217-79-6

**Table 6:** Heat map of top 25 binders of plastic chemicals bound to CAR, PXR, AR, PPARα, PPARγ, PPARδ, and RORγt. Light yellow represents compounds that appear twice between the NRs. Light orange represents compounds that appear three times. Dark orange represents compounds that appear 4 times. The darkest orange represents compounds appearing 5 times, and red represents compounds appearing in all 6 receptors.

	CAR	PXR	AR	PPARα	PPARγ	PPARδ	RORγt
1	5385-75-1	440102-72-7	56-49-5	5385-75-1	5385-75-1	128-80-3	1533-45-5
2	4076-39-5	5468-75-7	6925-69-5	57583-54-7	1324-76-1	54299-17-1	54299-17-1
3	57-97-6	7128-64-5	4076-39-5	24038-68-4	57583-54-7	1533-45-5	69227-22-1
4	24038-68-4	101463-69-8	57-97-6	3697-24-3	54299-17-1	128-69-8	177936
5	0376-06-07	54299-17-1	307-55-1	791156	1533-45-5	7128-64-5	1594-08-07
6	63-05-8	1650964	1705-85-7	72629-94-8	56-49-5	101463-69-8	72629-94-8
7	72629-94-8	24938-16-7	72629-94-8	0376-06-07	82657-04-03	5385-75-1	26375-23-5
8	307-55-1	5979-28-2	0376-06-07	2725-22-6	24038-68-4	56-49-5	0376-06-07
9	13595-25-0	1533-45-5	86112-79-0	56-49-5	101463-69-8	791156	7128-64-5
10	2772-45-4	22094-93-5	3697-24-3	1533-45-5	12237-62-6	1705-85-7	307-55-1
11	56-49-5	128-80-3	2058-94-8	1705-85-7	31107-36-5	24038-68-4	791156
12	6925-69-5	65181-78-4	7576-65-0	4129-84-4	177936	57583-54-7	3118-97-6
13	86112-79-0	31107-36-5	57-83-0	86112-79-0	26967-76-0	0376-06-07	0563-04-02
14	3697-24-3	68959-00-2	8003-22-3	23328-53-2	2398-96-1	18254-13-2	82657-04-03
15	1705-85-7	67905-17-3	302-97-6	57-97-6	4702-90-3	61788-44-1	302-97-6
16	2058-94-8	13676-91-0	63-05-8	177936	6505-28-8	177936	67905-17-3
17	84-15-1	5102-79-4	84-47-9	67905-17-3	90268-24-9	65181-78-4	7417-99-4
18	26898-17-9	981-40-8	335-76-2	4076-39-5	0376-06-07	68937-40-6	128-80-3
19	13676-91-0	18254-13-2	58-22-0	26967-76-0	6925-69-5	26967-76-0	31107-36-5
20	82657-04-03	61788-44-1	53-16-7	1324-76-1	78-32-0	72629-94-8	53-16-7
21	68411-46-1	4076-39-5	7128-64-5	12237-62-6	1330-78-5	57-97-6	68610-51-5
22	3910-35-8	0376-06-07	483-65-8	5522-43-0	13676-54-5	5468-75-7	8003-22-3
23	77-09-8	8003-22-3	5522-43-0	981-40-8	69227-22-1	67905-17-3	86112-79-0
24	57-41-0	177936	53-06-5	72363-26-9	128-80-3	82451-48-7	12237-62-6
25	54048-10-1	5124-25-4	2478-20-8	82-28-0	26898-17-9	4076-39-5	13676-91-0

**Table 7:** Heat map of docking scores of PFAS pesticides bound to CAR, AR, PXR, PPARα, PPARγ, PPARδ, and RORγt. The green color represents lower binding affinity and as the gradient approaches red, it indicates a higher binding affinity.

Compound	EPA ID	CAR	AR	PXR	PPARα	PPARγ	PPARδ	RORγt
Acifluorfen–sodium	114402	−8.8	−7.5	−8.5	−8	−8.9	−8.9	−9.2
Bifenthrin	128825	−9.6	−5.5	−10	−10.3	−10.1	−10.9	−9.5
Bromethalin	112802	−8.1	−7.2	−7.6	−5.7	−7.4	−5.8	−6.2
Chlorfenapyr	129093	−8.1	−7.8	−7.4	−6.4	−6.9	−7.6	−7.4
Cyflufenamid	555550	−9.1	−5.9	−9.4	−8.2	−10.1	−8.7	−7.4
Cyflumetofen	138831	−7.1	−5.9	−8.1	−6.1	−7.9	−7.5	−7.2
Dithiopyr	128994	−8.2	−7.1	−6.3	−5.2	−6	−7	−6.2
Ethalfluralin	113101	−8	−8	−6.9	−6	−7.1	−7	−6.8
Fipronil	129121	−7.7	−7.7	−8.2	−7.1	−7.3	−8	−8.4
Fluazifop-P-butyl	122809	−7.7	−7.8	−8.7	−8.3	−8.3	−8.9	−9
Flufenacet	121903	−8.1	−8.1	−8.7	−7.9	−8.4	−8.2	−8
Fluridone	112900	−9.3	−8	−9.5	−9.6	−10	−9.9	−9.2
Flurprimidol	125701	−8	−9.1	−8.5	−6.8	−8.4	−7.6	−7.1
Flutolanil	128975	−8.8	−5.5	−8.6	−7.9	−8.5	−9.2	−9.3
Fluvalinate	109302	−8.7	#N/A	−10.4	−9.7	−10.3	−10.6	−10
Fomesafen	123802	#N/A	−7.1	#N/A	#N/A	#N/A	#N/A	#N/A
gamma-Cyhalothrin	128807	−10	−6.4	−9.7	−9	−9.3	−10.2	−7.8
Hexaflumuron	118202	−7.1	−7.1	#N/A	−9.6	−8.9	−9.8	−9.6
Hydramethylnon	118401	−7.1	−8.2	−10.4	−9.6	−10.2	−12.3	−8.9
Lactofen	128888	−8.1	−7	−9.1	−9.1	−9	−9.2	−8.7
lambda-Cyhalothrin	128897	#N/A	−8.8	−9.9	−8	−9.9	−10	−6.5
Norflurazon	105801	−8.4	−7.6	−8.3	−7.8	−8.3	−8.2	−8
Novaluron	124002	−7.4	−6.6	−10.7	−9.5	−10.3	−10.5	−10.4
Noviflumuron	118204	−7.8	−9.3	−10.8	−9.6	−9.4	−10.4	−10.8
Oxathiapiprolin	128111	−8	−9	−10.7	−9.2	−10.8	−11.6	−10.5
Oxyfluorfen	111601	−8.2	−6.1	−8.3	−7.9	−8.5	−8.6	−8.6
Penoxsulam	119031	−8.1	−7.3	−9.2	−7.7	−9	−7.7	−9.1
Picoxystrobin	129200	−9.4	−7.1	−8.3	−8.3	−7.7	−8.5	−7.4
Prodiamine	110201	−7.3	−5.9	−6.6	−5.9	−6.6	−6.6	−6.2
Prosulfuron	129031	−8.1	−9.5	−9.6	−8.4	−10	−8.9	−9.2
Pyridalyl	295149	−8.9	−8.5	−9.4	−8.5	−8.7	−9.3	−9.2
Pyrifluquinazon	555555	−9.2	−6.2	−9.7	−8.2	−10.3	−9.9	−8.5
Saflufenacil	118203	−6.6	−8.8	−10.6	−8.4	−9	−10.4	−8.9
Tefluthrin	128912	−9.2	−8	−9	−7.9	−9	−9.5	−9.9
Tetraconazole	120603	−8.1	−8.1	−8.1	−7.1	−7.9	−7.4	−7.8
Tralopyril	119093	−8.9	−8.2	−8	−7.2	−8.7	−7.9	−8.1
Trifloxystrobin	129112	−9.2	−7.3	−9.8	−9.2	−9.1	−8.8	−9.4
Triflumizole	128879	−8.7	−6.8	−7	−7.6	−7.3	−7.2	−7.6
Triflusulfuron-methyl	129002	−6.8	#N/A	−9.6	−8.9	−9.8	−8.8	−9

**Table 8:** Heat map of docking scores of bisphenols bound to CAR, AR, PXR, PPARα, PPARγ, PPARδ, and RORγt. The green color represents lower binding affinity and as the gradient approaches red, it indicates a higher binding affinity.

Compound	CASRN	CAR	AR	PXR	PPARα	PPARγ	PPARδ	RORγt
4,4′-isopropylidenediphenol	80-05-7	−8.6	−8.2	−8.4	−6.9	−7	−7.5	−7
4,4′-sulphonyldiphenol	80-09-1	−7.8	−7.6	−7.6	−6.7	−7	−7.6	−7
4,4′-methylenediphenol	620-92-8	−7.5	−7.4	−7.5	−7.2	−7.7	−7	−7.7
4,4′-(1-methylpropylidene)bisphenol	77-40-7	−8.5	−7.9	−8.4	−6.9	−7	−7.7	−7
4,4′-[2,2,2-trifluoro-1-(trifluoromethyl)ethylidene]diphenol	1478-61-1	−8.7	−7.8	−9.7	−7.1	−7.3	−7.6	−7.3
4,4′-(1,3-phenylene-bis(1-methylethylidene))bisphenol	13595-25-0	−11.2	−8.3	−9.4	−7.1	−9.9	#N/A	#N/A
4,4′-(1-Phenylethylidene)bisphenol	1571-75-1	−9.2	−7.2	−7.6	−6.4	−7.7	−8.8	−7.7
9,9-Bis(4-hydroxyphenyl)fluorene	3236-71-3	−6.7	−6	−8.4	−7.5	−8	−7	−8
Biphenyl-4,4′-diol	92-88-6	−7.5	−7.5	−7.2	−6.8	−7.3	−6.9	−7.3
4,4′-isopropylidenedi-o-cresol	79-97-0	−8.9	#N/A	−9	−7	−8	−8	−8
4,4′-(dichlorovinylidene)diphenol	14868-03-02	−7.9	−8.1	−7.7	−5.9	−7.3	−7.7	−7.3
4,4′-(1,4-Phenylenediisopropylidene)bisphenol	2167-51-3	−7.2	−6.5	−9.5	−8.6	−8.7	−8.9	−8.7
4,4′-cyclohexylidenebisphenol	843-55-0	−8.8	−7.6	−8.3	−6.7	−6.5	−8.2	−6.5
2,2-bis(2-hydroxy-5-biphenylyl)propane	24038-68-4	−11.5	−6.8	−9.6	−10.3	−9.8	−11.1	−9.8
4,4′-ihydroxytetraphenylmethane	1844-01-05	−5.9	−5.6	−8.3	−6.5	−7.7	−8.7	−7.7
4,4′-(1,3-phenylene-bis(1-methylethylidene))bis-phenol	13595-25-0	−11.2	−8.3	−9.4	−7.1	−9.9	#N/A	#N/A
9,9-Bis(4-hydroxyphenyl)fluorene	3236-71-3	−6.7	−6	−8.4	−7.5	−8	−7	−8
4,4′-cyclohexylidenebisphenol	843-55-0	−8.8	−7.6	−8.3	−6.7	−6.5	−8.2	−6.5
Biphenyl-4,4′-diol	92-88-6	−7.5	−7.5	−7.2	−6.8	−7.3	−6.9	−7.3

**Table 9:** Heat map of docking scores of phthalates bound to CAR, AR, PXR, PPARα, PPARγ, PPARδ, and RORγt. The green color represents lower binding affinity and as the gradient approaches red, it indicates a higher binding affinity.

Compound	CASRN	CAR	AR	PXR	PPARα	PPARγ	PPARδ	RORγt
Dimethyl phthalate	131-11-3	#N/A	#N/A	−6.7	#N/A	#N/A	#N/A	#N/A
Diethyl phthalate	84-66-2	−6.7	−6.3	−6.8	−6.3	−6.7	−6.1	−6.6
Diallyl phthalate	131-17-9	−7.2	−6.5	−6.9	−6.7	−7.5	−6.6	−6.6
Di-n-propyl phthalate	131-16-8	−7.2	−6.4	−6.8	−6.5	−7.3	−6.3	−6.4
Di-n-butyl phthalate	84-74-2	−6.8	−6.9	−6.7	−6.9	−7.1	−6.7	−7.3
Diisobutyl phthalate	84-69-5	−7.7	−7.3	−7.3	−7	−7.6	−7	−7.3
Di-2-methoxyethyl phthalate	117-82-8	−6.6	−6.2	−6.7	−6.5	−6.9	−6	−6.3
Butyl cyclohexyl phthalate	84-64-0	−8	−7.9	−7.9	−7.5	−8.1	−7.8	−8
Di-n-pentyl phthalate	131-18-0	−7.4	−7	−7.2	−6.8	−7.5	−7.2	−5.4
Dicyclohexyl phthalate	84-61-7	−9.7	−8.7	−8.6	−8.6	−9	−9.3	−9.4
Butyl benzyl phthalate	85-68-7	−8.4	−7.8	−8.2	−8.2	−8.8	−8.3	−7.9
Di-n-hexyl phthalate	84-75-3	−6.9	−7.1	−7	−6.9	−7.2	−7.4	−7.6
Diisohexyl phthalate	146-50-9	−8.2	−7.3	−7.7	−7.4	−8.2	−7.9	−7.6
Diisoheptyl phthalate	41451-28-9	−8	−7	−8.1	−7.2	−8	−8.2	−8
Butyl decyl phthalate	89-19-0	−7.9	−6.9	−7.4	−7.3	−7.5	−7.4	−7.5
Dibutoxy ethyl phthalate	117-83-9	−6.9	−7	−7	−7.3	−7.1	−7.2	−7.4
Di(2-ethylhexyl) phthalate	117-81-7	−8	−5.9	−8	−7.5	−7.8	−7.9	−8.5
Di(n-octyl) phthalate	117-84-0	−8	−7.3	−7.3	−7.5	−7.7	−7.7	−7.8
Diisooctyl phthalate	27554-26-3	−8.2	−7.6	−8	−7.4	−7.8	−8.2	−8.3
n-Octyl n-decyl phthalate	119-07-3	#N/A	#N/A	−7.3	#N/A	#N/A	#N/A	#N/A
Diisononyl phthalate	28553-12-0	−7.9	−8	−7.7	−7.5	−7.6	−8.3	−8
Di(2-propylheptyl) phthalate	53306-54-0	−7.9	−6.9	−7.9	−7.5	−7.8	−7.8	−8.3
Diisodecyl phthalate	26761-40-0	−7.6	−7.5	−8	−7.9	−7.7	−8.1	−8.5
Diundecyl phthalate	3648-20-2	−6.7	−7.4	−7.1	−7.3	−7.4	−7.7	−7.8
Diisoundecyl phthalate	85507-79-5	−7.7	−7.3	−8.3	−7.8	−7.7	−8	−8.5
Ditridecyl phthalate	119-06-2	#N/A	#N/A	−6.9	#N/A	#N/A	#N/A	#N/A
Diisotridecyl phthalate	68515-47-9	−7.4	−7.3	−7.5	−8	−8	−8.3	−8.2

**Table 10:** Binding affinity of nuclear receptors tested with DCHP and DEHP. The functional activity column displays the activation assay results.

Receptors	DEHP (CAS ID:117-81-7) Binding Affinity (kcal/mol)	DEHP Functional Activity	DCHP (CAS ID: 84-61-7) Binding Affinity (kcal/mol)	DCHP Functional Activity
PXR	−8.0	Agonist	−8.6	Agonist
CAR	−8.0	Agonist	−9.7	Agonist
PPARα	−7.5	Agonist	−8.6	Antagonist
PPARγ	−7.8	Agonist	−9.0	Agonist
PPARδ	−7.9	Antagonist	−9.3	Neutral
AR	−5.9	Agonist	−8.7	Agonist
RORγt	−8.5	Neutral	−9.4	Antagonist
RARγ	−8.4	Antagonist	−9.9	Antagonist
RARβ	−5.5	Antagonist	−9.9	Antagonist
RORβ	−7.8	Antagonist	−8.8	Antagonist
RXRβ	−7.6	Antagonist	−8.7	Agonist
SF-1	−6.8	Neutral	−9.0	Antagonist
ERα	−7.1	Agonist	−8.6	Agonist

## Data Availability

Data are contained within the article or Supplementary Materials.

## Supplementary Materials

The supporting information can be downloaded at https://www.mdpi.com/article/10.3390/ijms26072879/s1.
