# Supplementary material for: Impact of Persistent Endocrine-Disrupting Chemicals on Human Nuclear Receptors: Insights from In Silico and Experimental Characterization
Source: Int J Mol Sci. 2025 Mar 21;26(7):2879. doi: 10.3390/ijms26072879 (PMC11988381; doi:10.3390/ijms26072879)
Supplement: Supplementary file 1 [file ijms-26-02879-s001.zip › Suppl. Tables-Figures/Supplementary Figures.pdf]

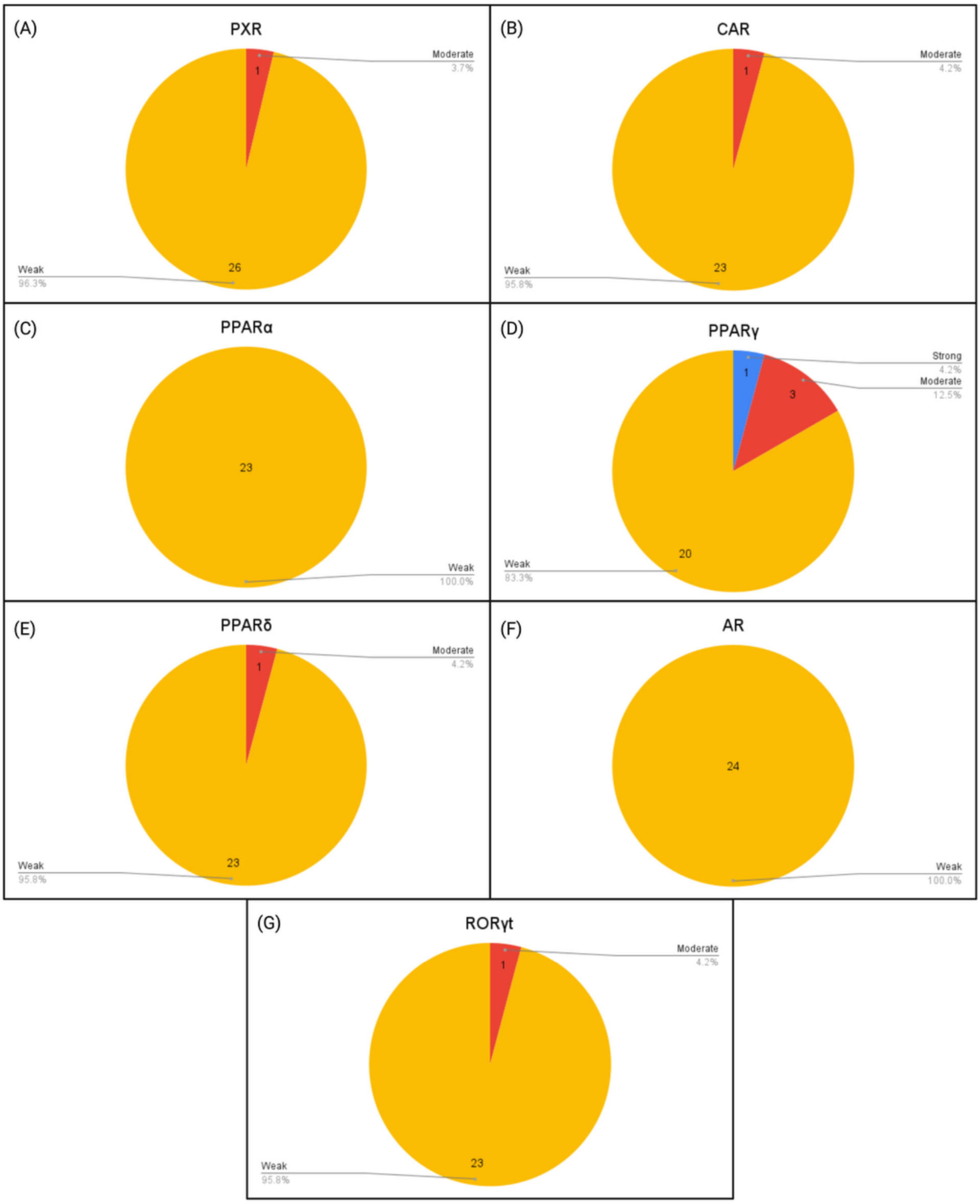

Supplementary Figure S1. Percentage of NRs strong, moderate, and weak phthalate binders with count **(A)** PXR **(B)** CAR **(C)** PPARα **(D)** PPARγ **(E)** PPARδ **(F)** AR **(G)** RORγt

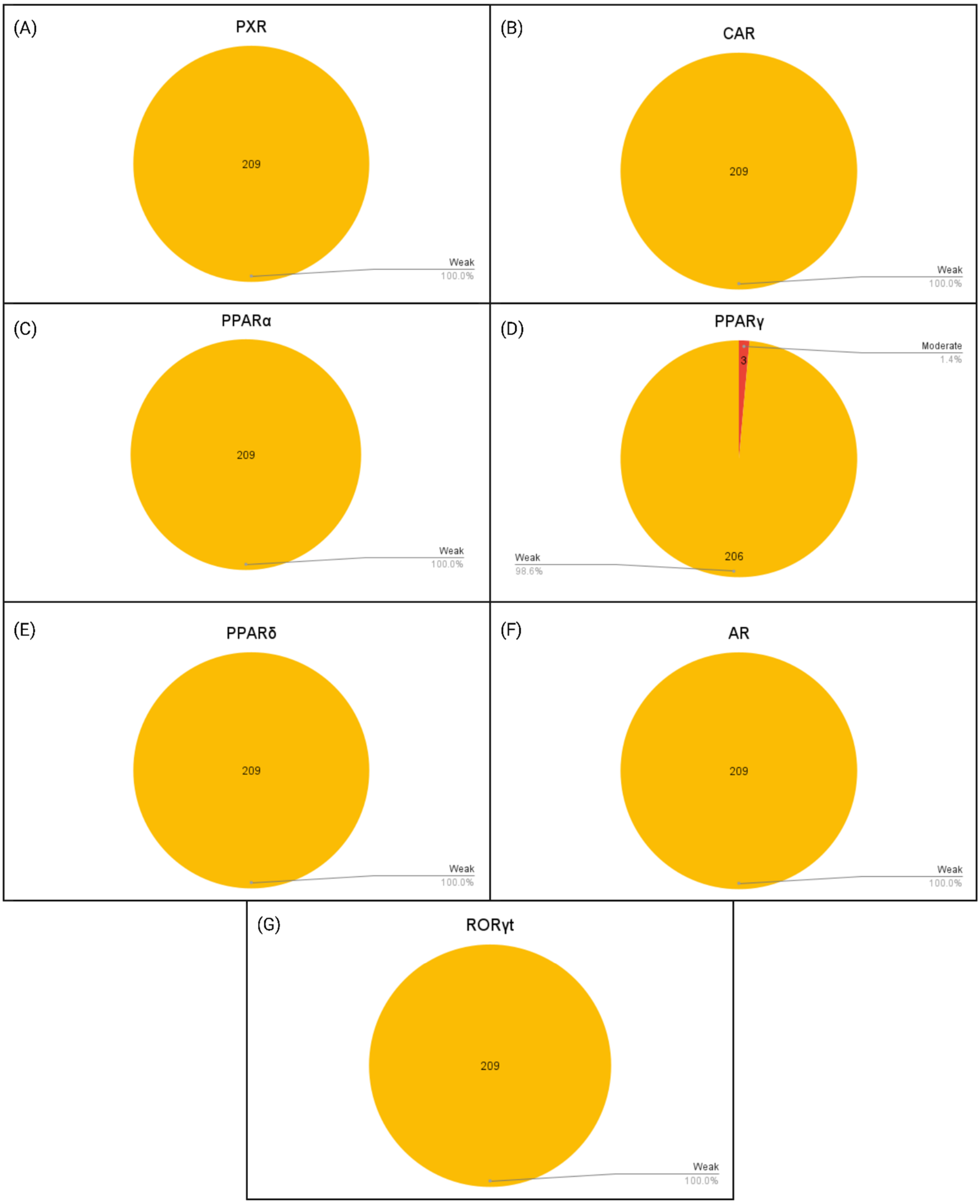

Supplementary Figure S2. Percentage of NRs strong, moderate, and weak PBDE binders with count. **(A)** PXR **(B)** CAR **(C)** PPARα **(D)** PPARγ **(E)** PPARδ **(F)** AR **(G)** RORγt

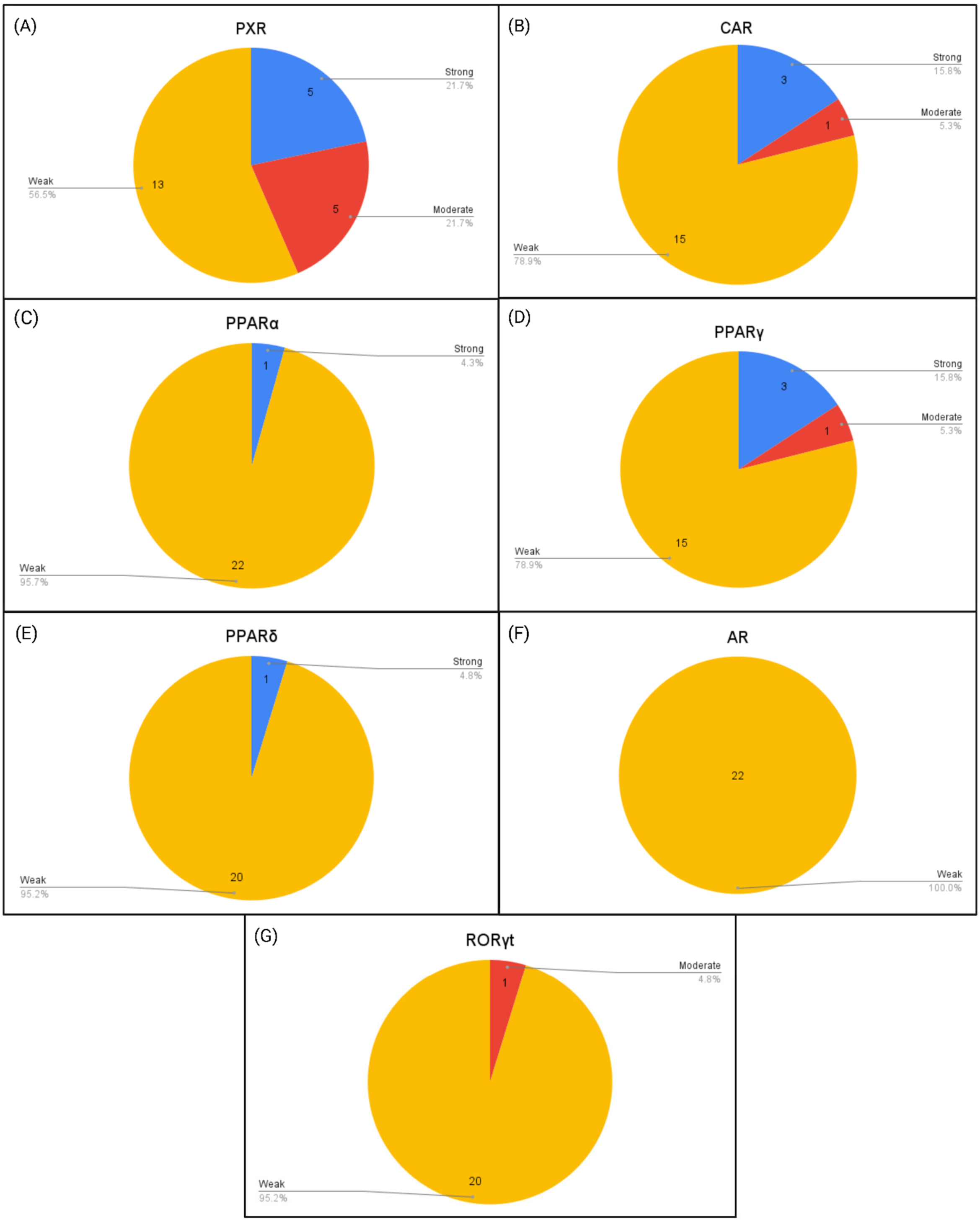

Supplementary Figure S3. Percentage of NRs strong, moderate, and weak bisphenol binders with count **(A)** PXR **(B)** CAR **(C)** PPARα **(D)** PPARγ **(E)** PPARδ **(F)** AR **(G)** RORγt

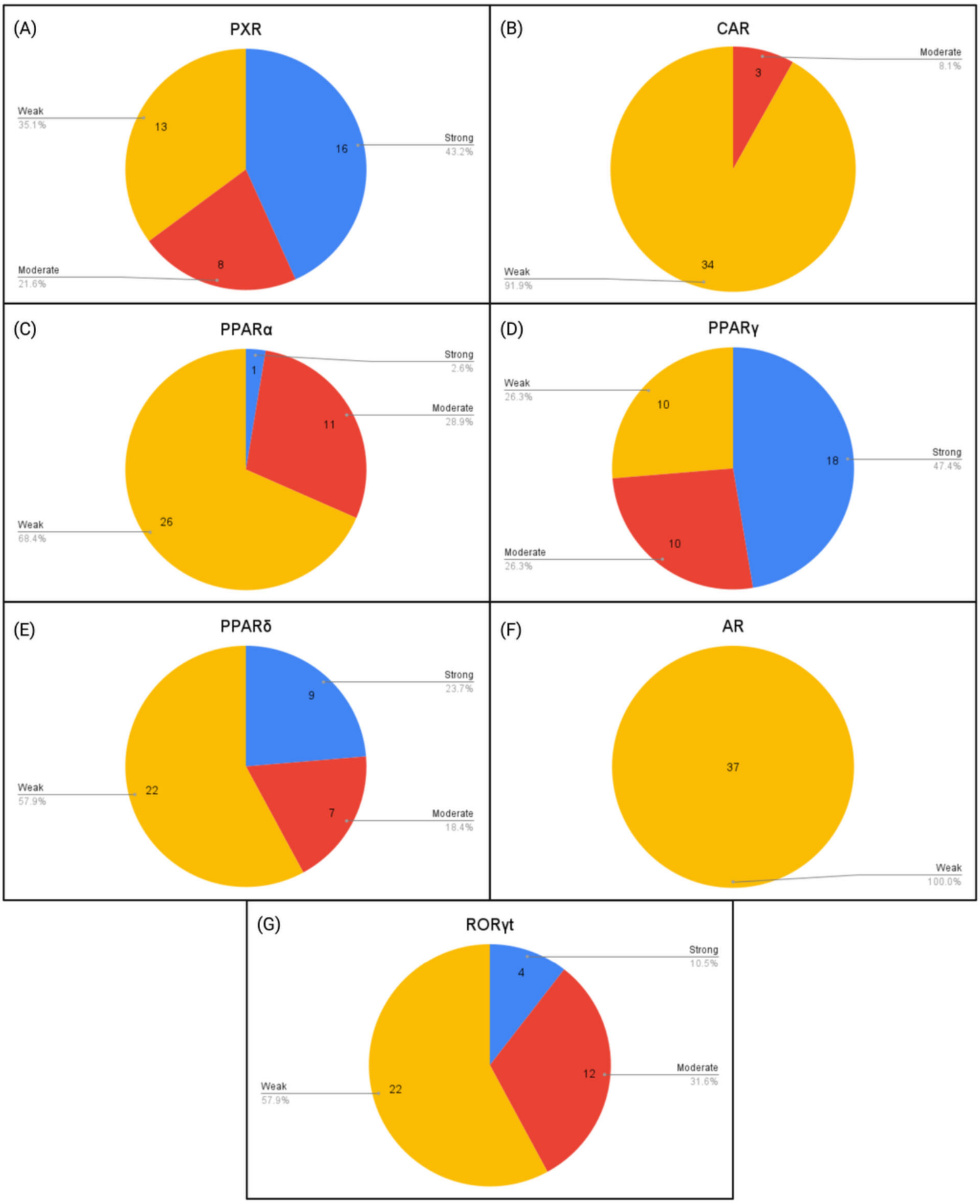

Supplementary Figure S4. Percentage of NRs strong, moderate, and weak PFAS pesticide binders with count **(A)** PXR **(B)** CAR **(C)** PPARα **(D)** PPARγ **(E)** PPARδ **(F)** AR **(G)** RORγt

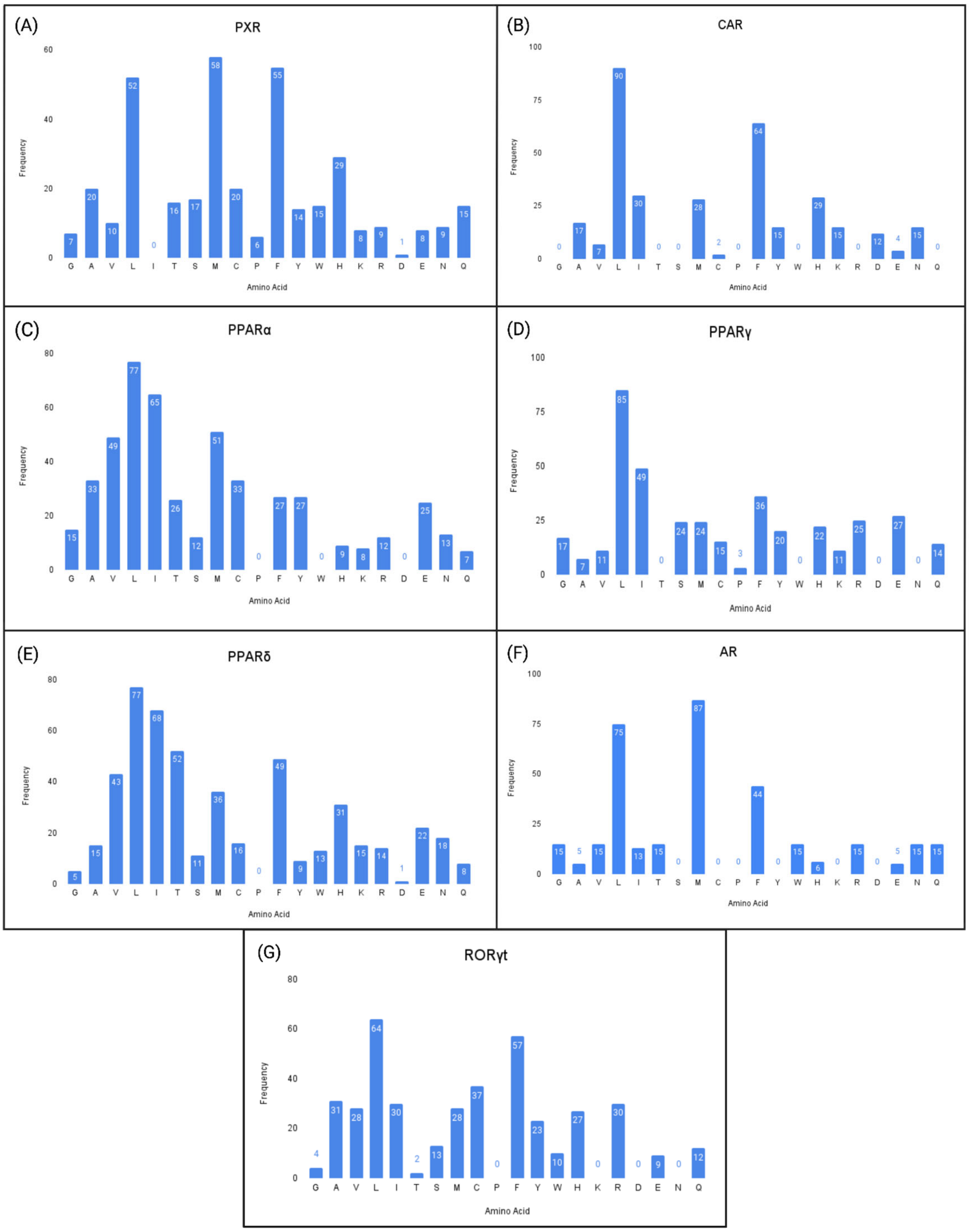

Supplementary Figure S5. Distribution of NRs amino acid residues interacting with top 15 PFAS binders **(A)** PXR **(B)** CAR **(C)** PPARα **(D)** PPARγ **(E)** PPARδ **(F)** AR **(G)** RORγt

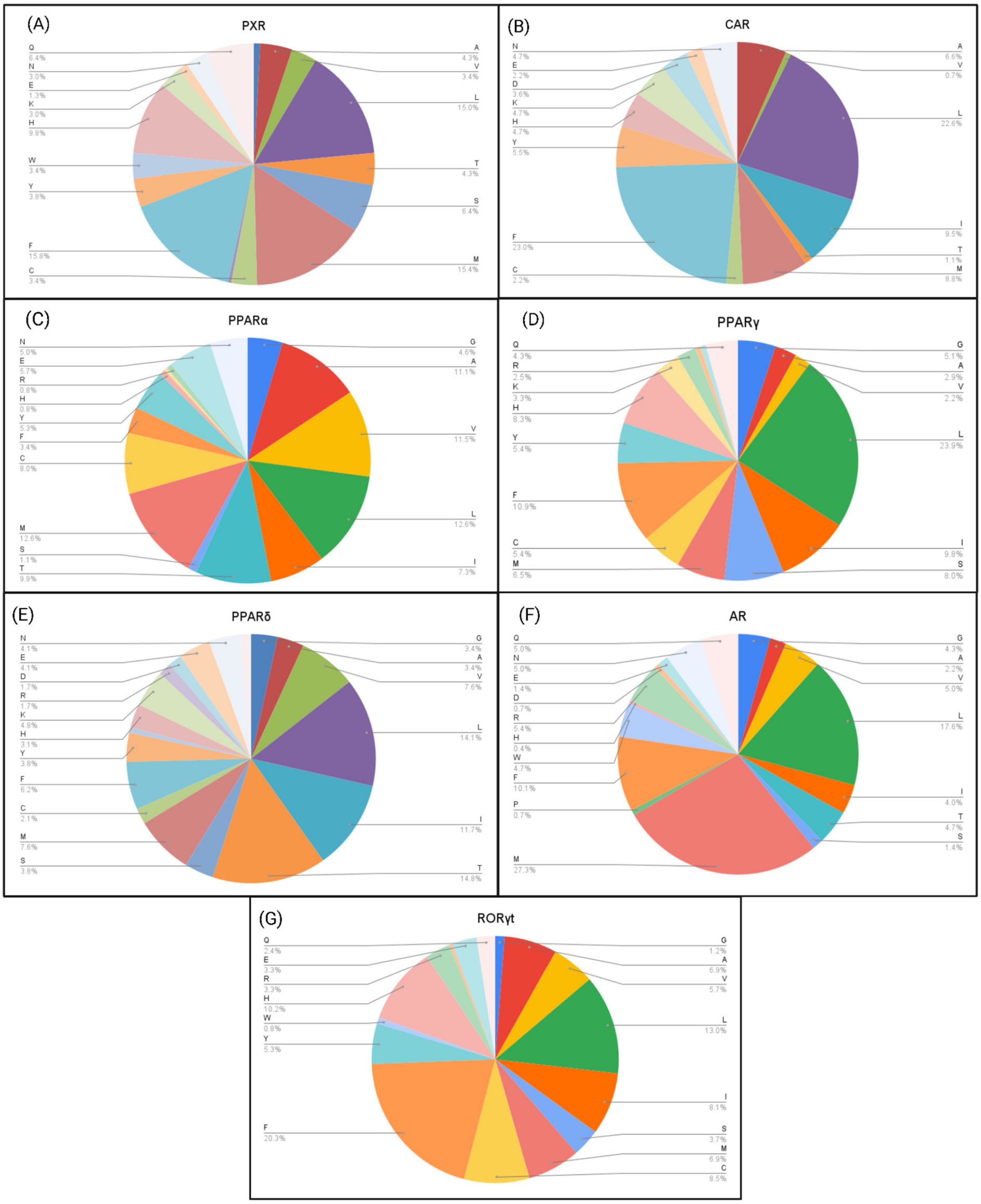

Supplementary Figure S6. Percentage of NRs amino acid residues interacting with top 15 bisphenol binders **(A)** PXR **(B)** CAR **(C)** PPARα **(D)** PPARγ **(E)** PPARδ **(F)** AR **(G)** RORγt

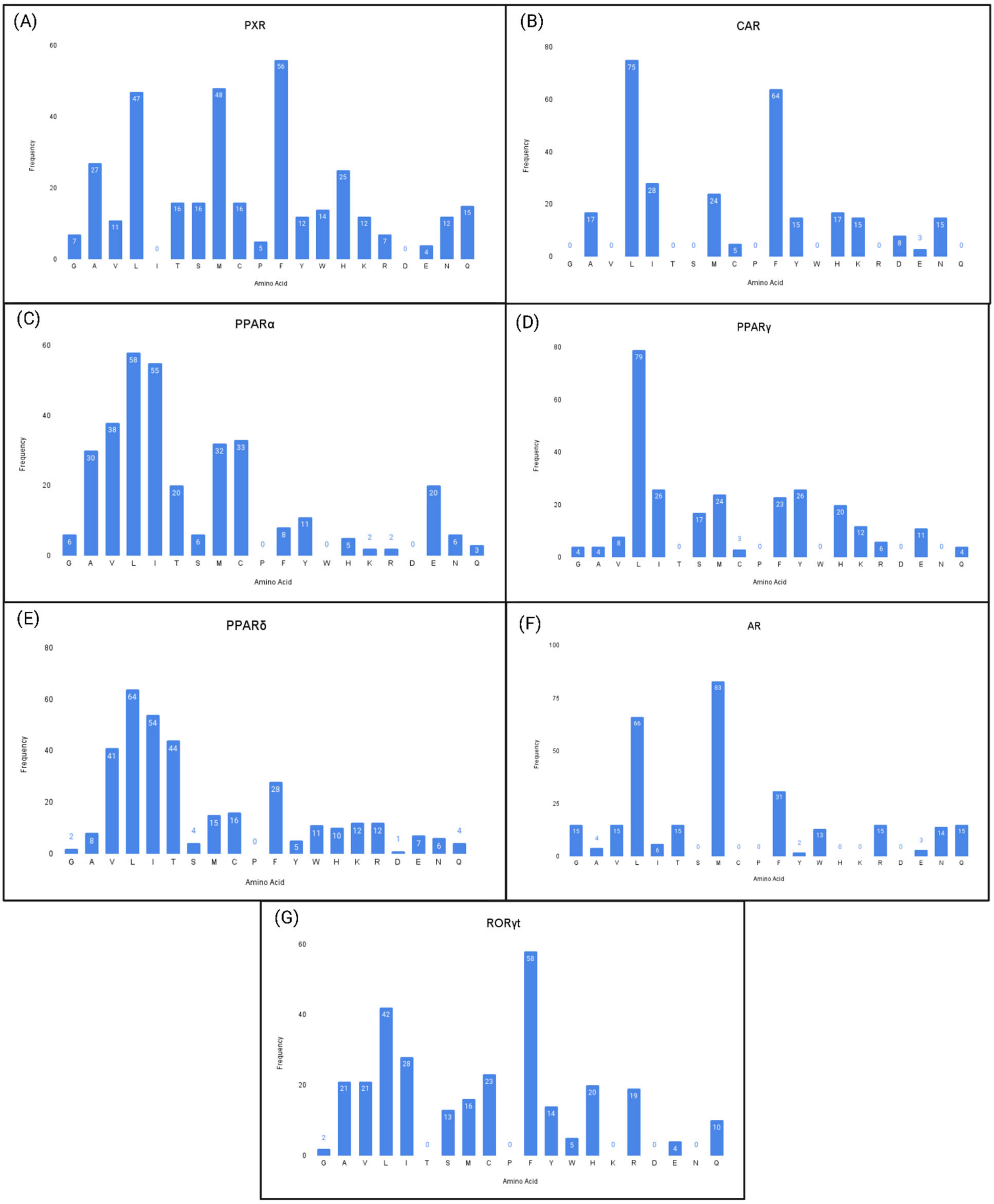

Supplementary Figure S7. Distribution of NRs amino acid residues interacting with top 15 nanoplastic binders **(A)** PXR **(B)** CAR **(C)** PPARα **(D)** PPARγ **(E)** PPARδ **(F)** AR **(G)** RORγt

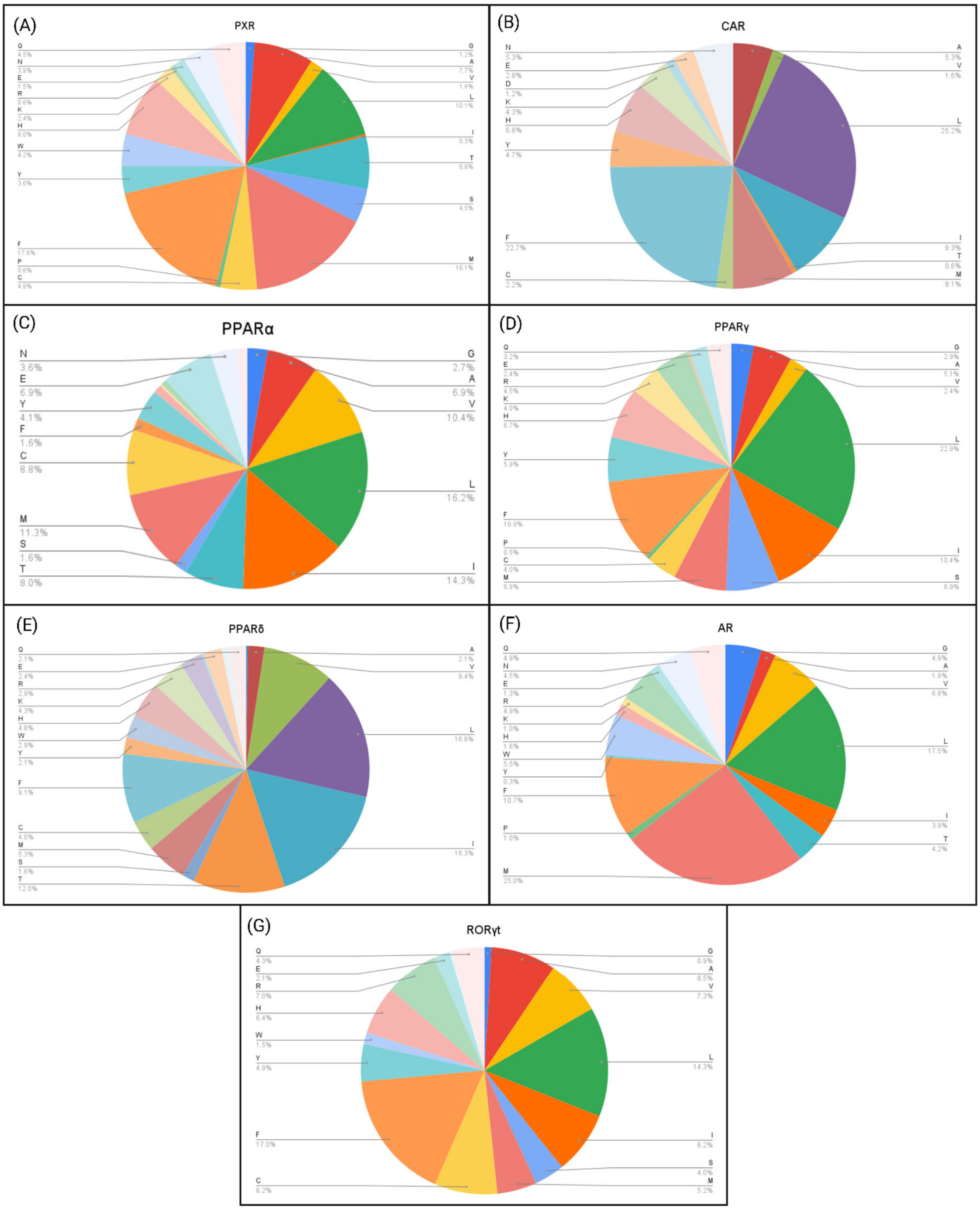

Supplementary Figure S8. Percentage of NRs amino acid residues interacting with top 15 PFAS pesticide binders **(A)** PXR **(B)** CAR **(C)** PPARα **(D)** PPARγ **(E)** PPARδ **(F)** AR **(G)** RORγt

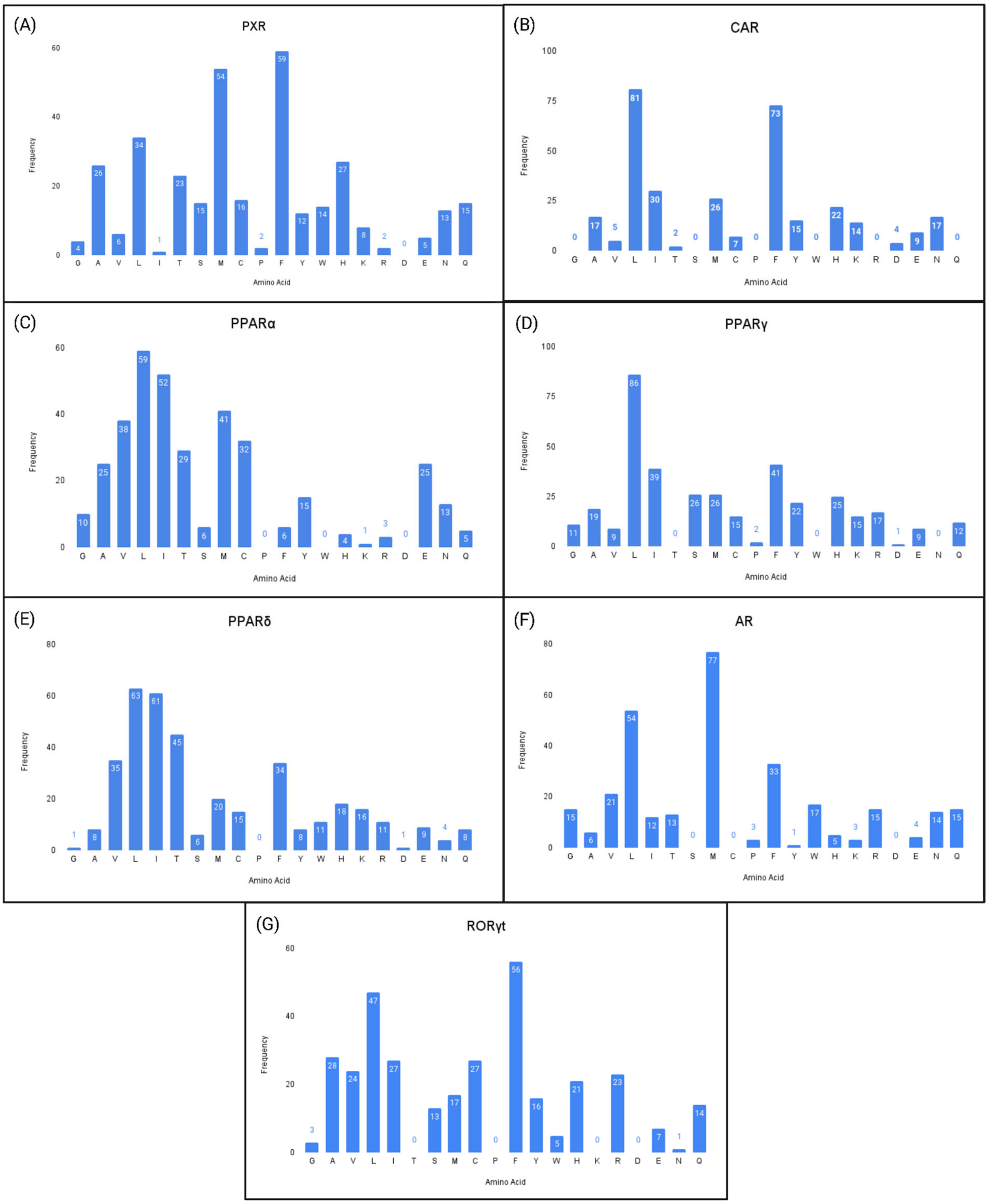

Supplementary Figure S9. Distribution of NRs amino acid residues interacting with top 15 PFAS pesticide binders. **(A)** PXR **(B)** CAR **(C)** PPARα **(D)** PPARγ **(E)** PPARδ **(F)** AR **(G)** RORγt

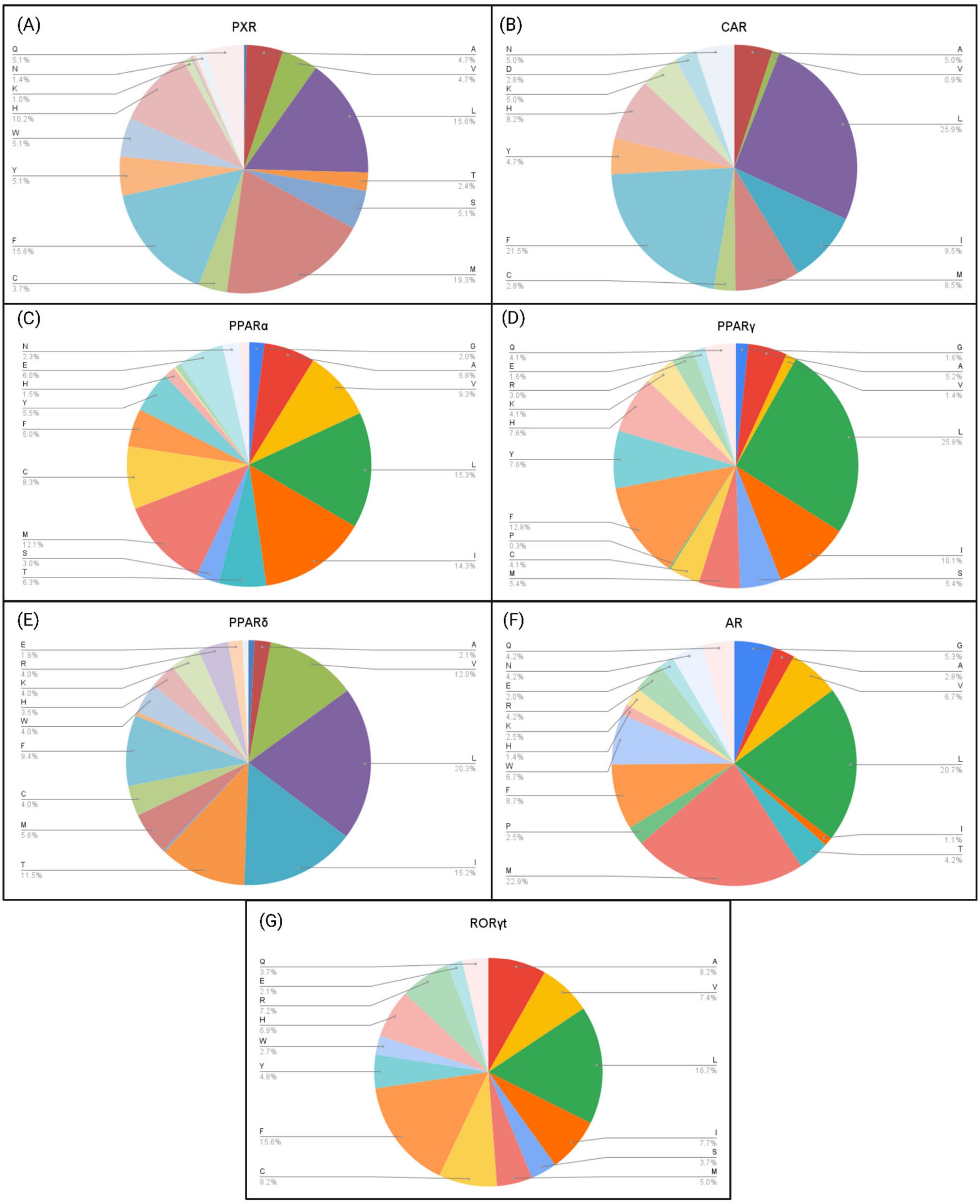

Supplementary Figure S10. Percentage of NRs amino acid residues interacting with top 15 phthalate binders. **(A)** PXR **(B)** CAR **(C)** PPARα **(D)** PPARγ **(E)** PPARδ **(F)** AR **(G)** RORγt

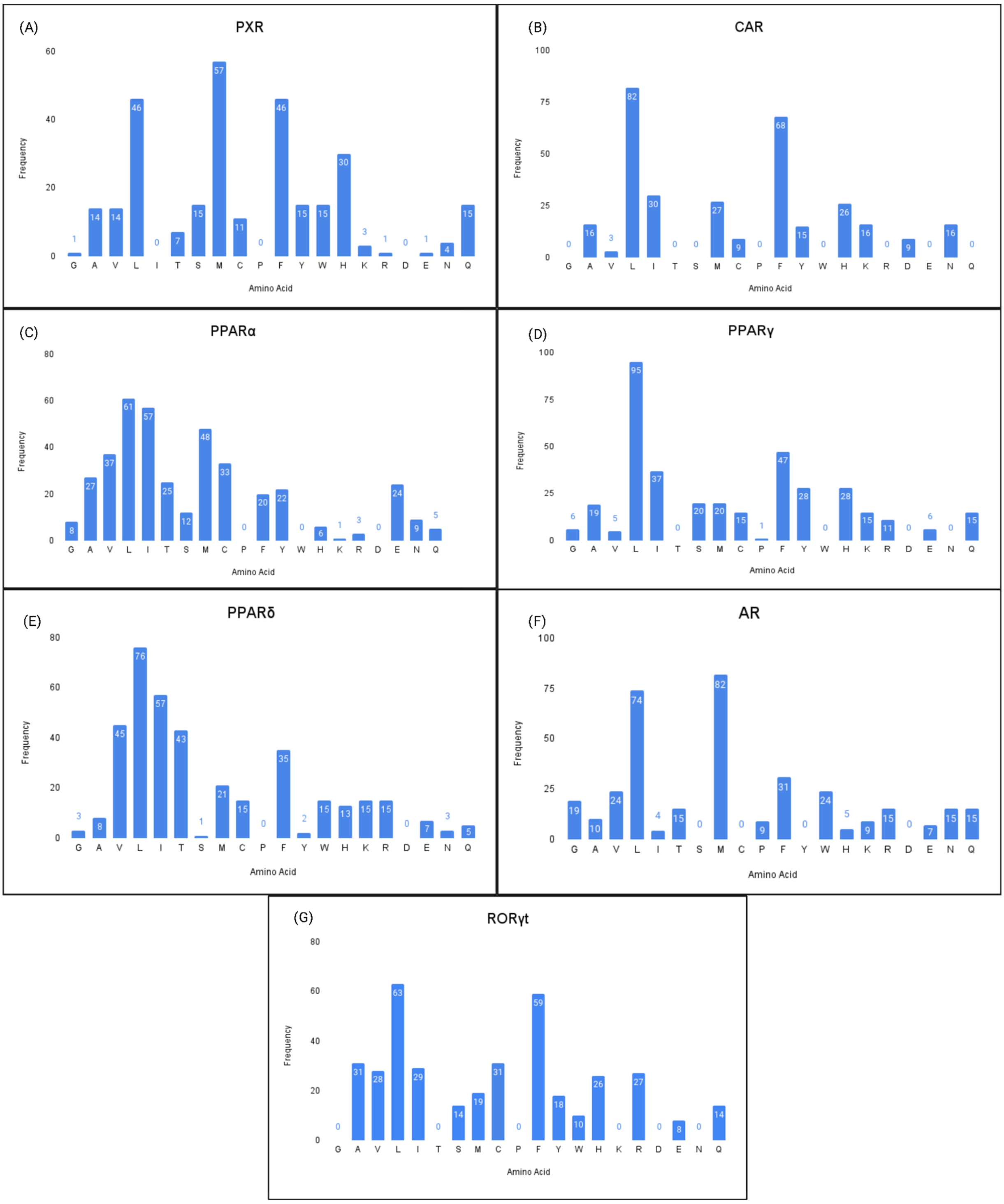

Supplementary Figure S11. Distribution of NRs amino acid residues interacting with top 15 phthalate binders. **(A)** PXR **(B)** CAR **(C)** PPARα **(D)** PPARγ **(E)** PPARδ **(F)** AR **(G)** RORγt

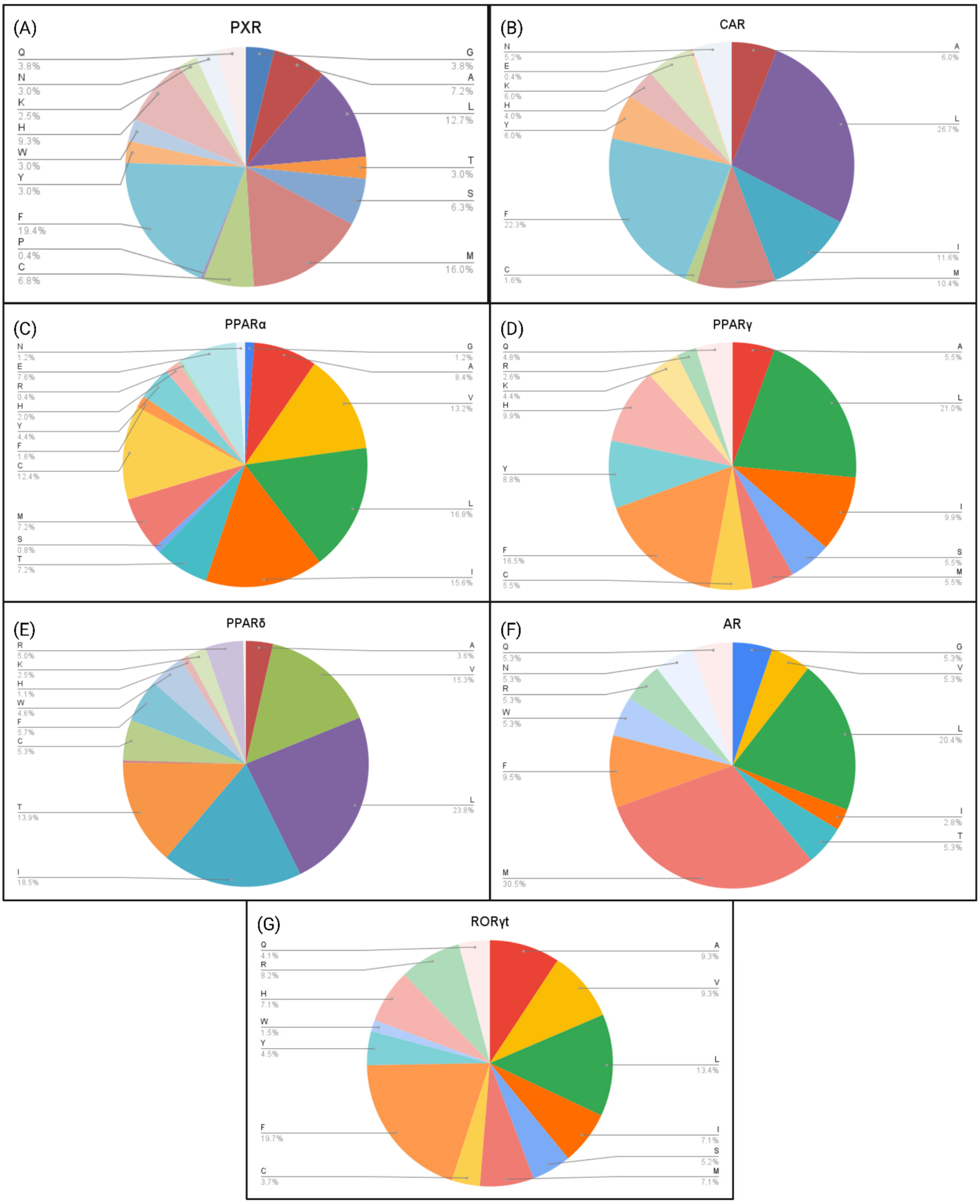

Supplementary Figure S12. Percentage of NRs amino acid residues interacting with top 15 PBDE binders. **(A)** PXR **(B)** CAR **(C)** PPARα **(D)** PPARγ **(E)** PPARδ **(F)** AR **(G)** RORγt

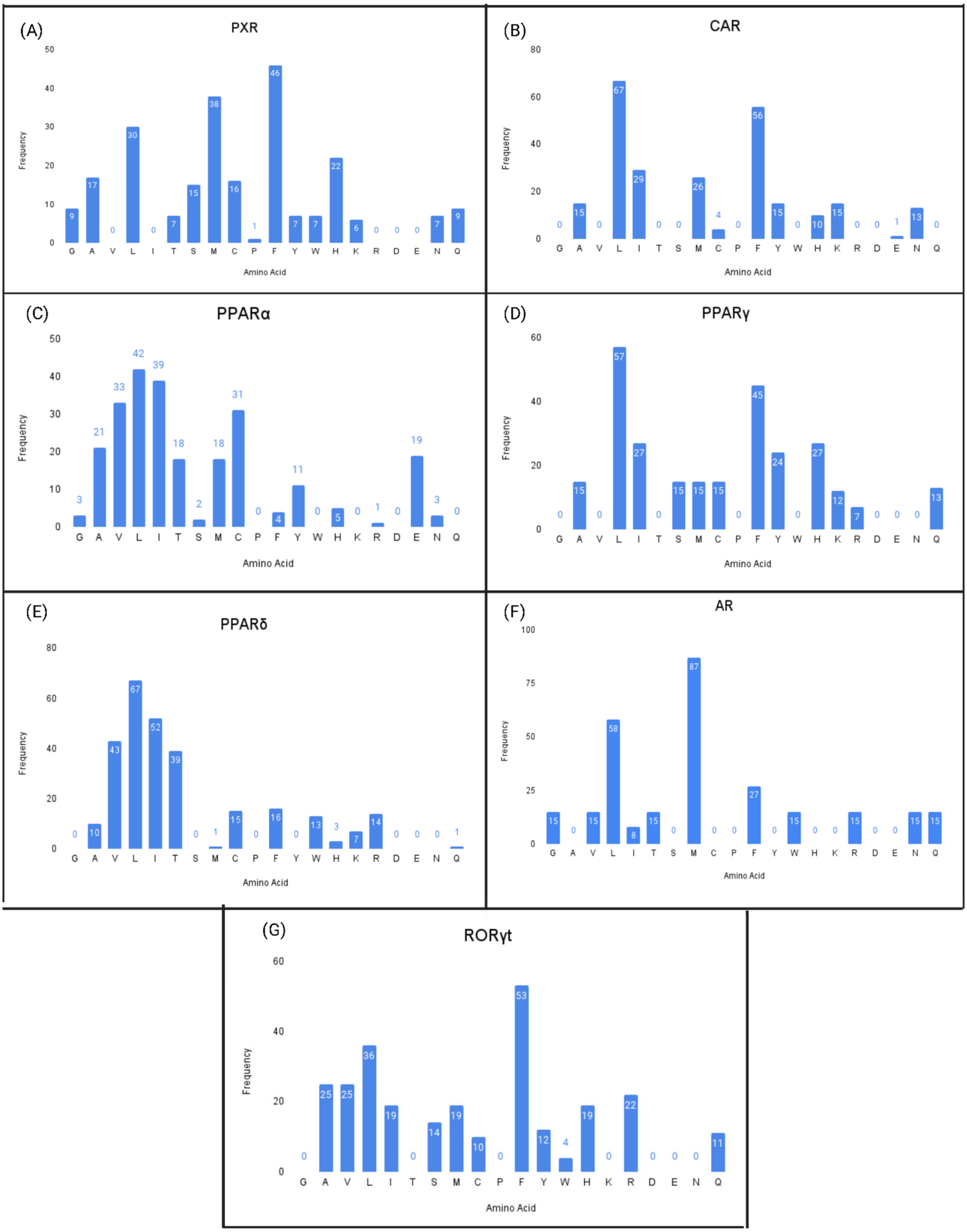

Supplementary Figure S13. Distribution of NRs amino acid residues interacting with top 15 PBDE binders. **(A)** PXR **(B)** CAR **(C)** PPARα **(D)** PPARγ **(E)** PPARδ **(F)** AR **(G)** RORγt

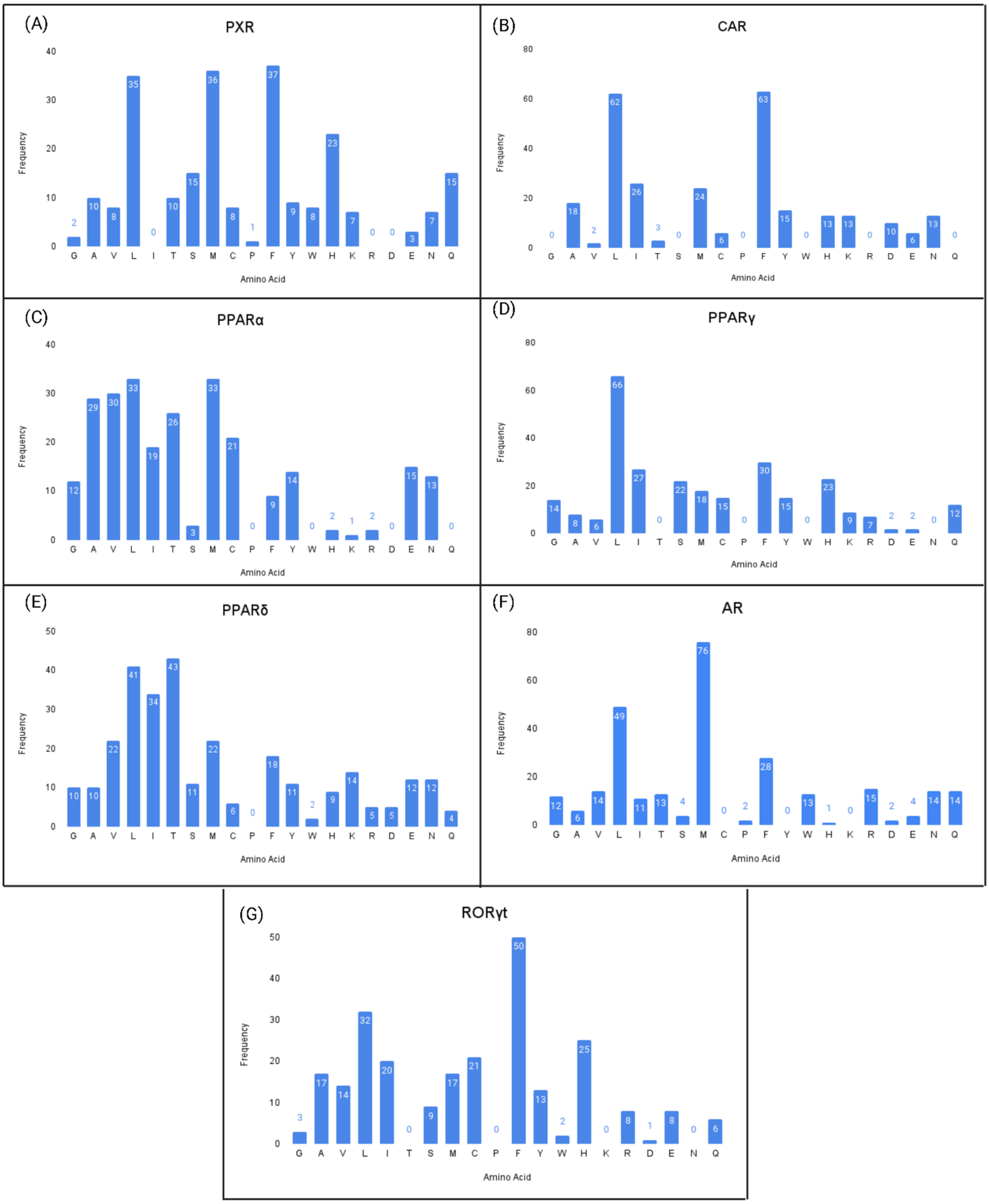

Supplementary Figure S14. Distribution of NRs amino acid residues interacting with top 15 bisphenol binders. **(A)** PXR **(B)** CAR **(C)** PPARα **(D)** PPARγ **(E)** PPARδ **(F)** AR **(G)** RORγt

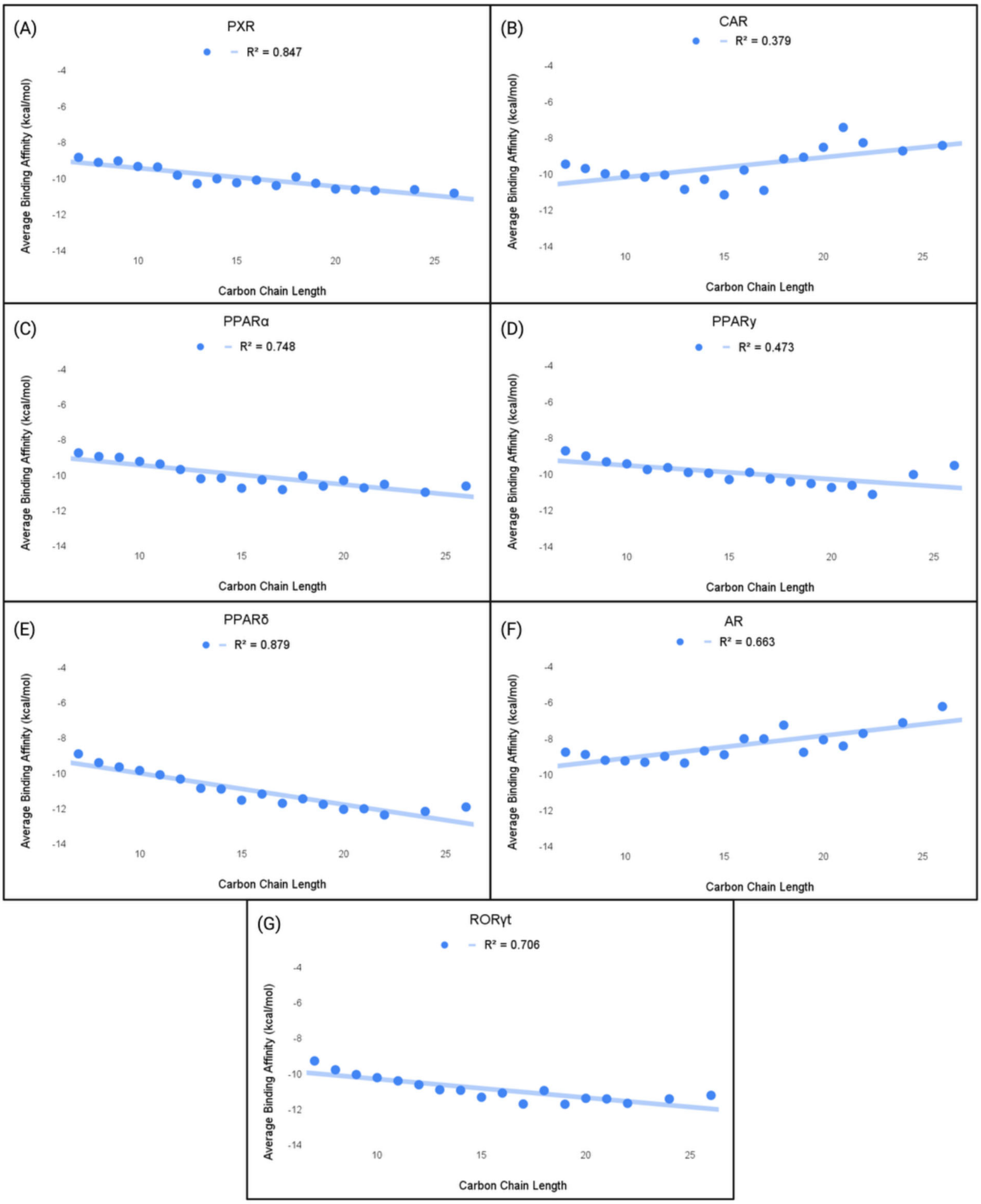

Supplementary Figure S15. Long chain length vs average binding affinity of PFAS to NRs **(A)** PXR **(B)** CAR **(C)** PPARα **(D)** PPARγ **(E)** PPARδ **(F)** AR **(G)** RORγt

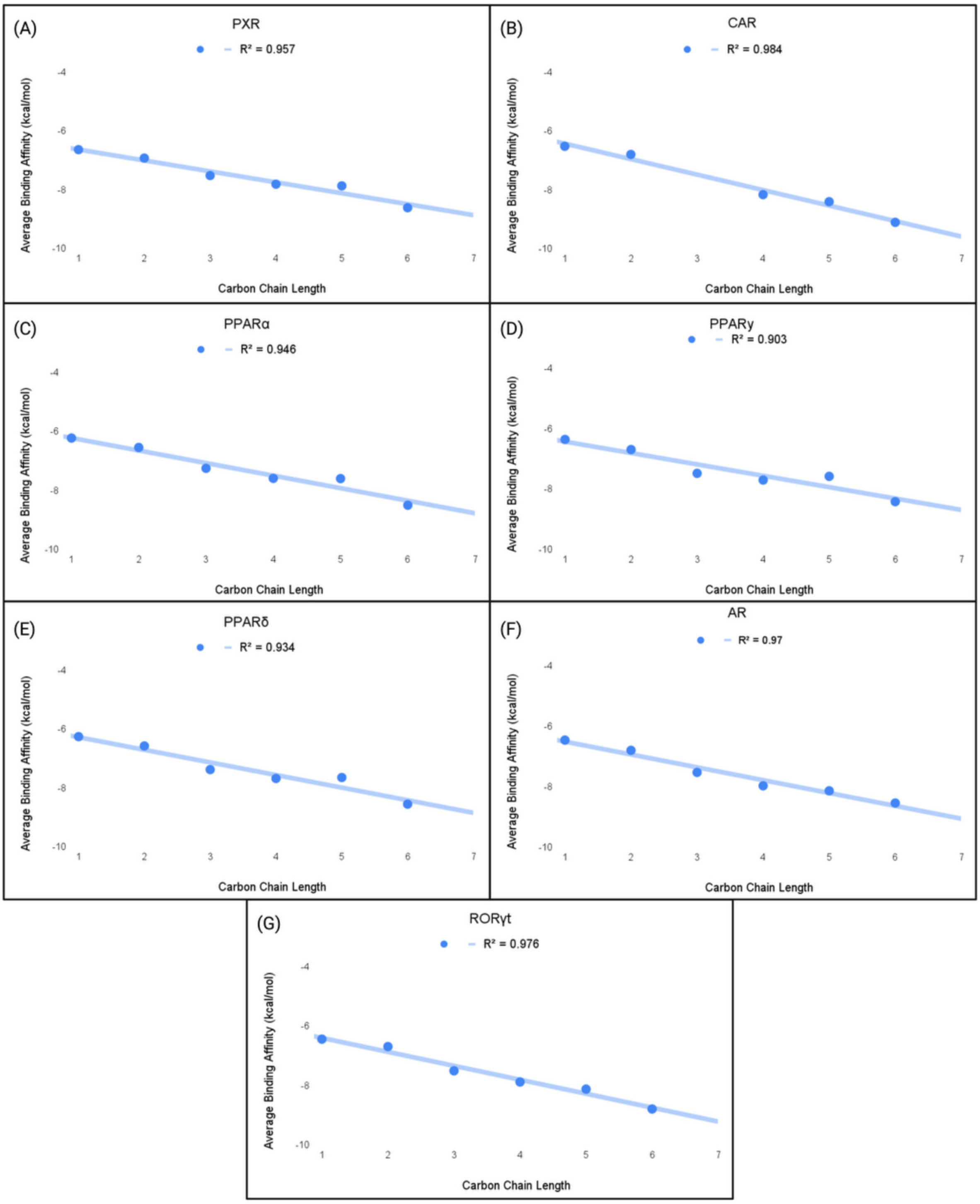

Supplementary Figure S16. Short chain length vs average binding affinity of PFAS to NRs **(A)** PXR **(B)** CAR **(C)** PPARα **(D)** PPARγ **(E)** PPARδ **(F)** AR **(G)** RORγt

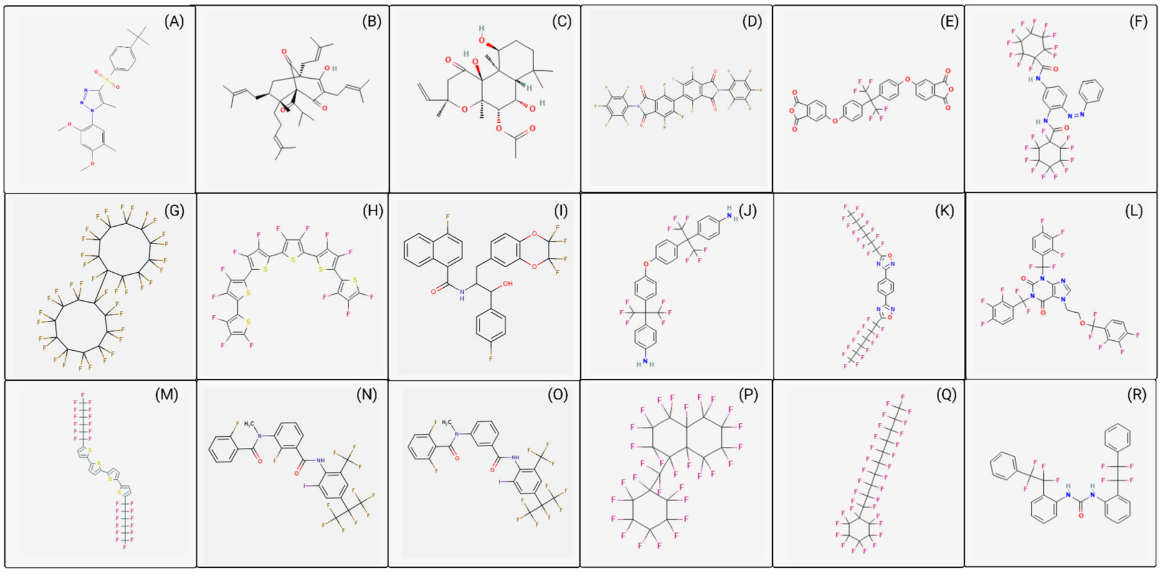

Supplementary Figure S17. 2D Structures of the Top 3 Endogenous Ligands and Top 15 PFAS bound to PXR. **(A)** 2135395-15-0 **(B)** 11079-53-1 **(C)** 66575-29-9 (D) 5366-99-4 (E) 61778-79-8 (F) 548470-06-0 (G) NOCAS_871520 (H) 347838-21-5 (I) NOCAS_1026885 (J) 131662-80-1 (K) 146304-71-4 (L) 126565-13-7 (M) 446043-85-2 (N) NOCAS_1026915 (O) NOCAS_1026998 (P) 125061-94-1 (Q) 87667-00-3 (R) 61547-75-9

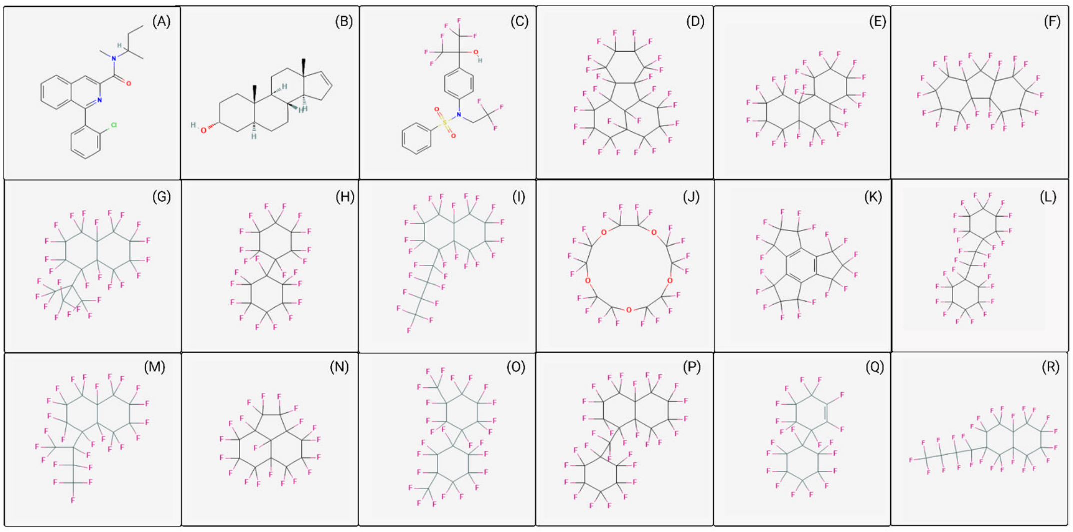

Supplementary Figure S18. 2D Structures of the Top 3 Endogenous Ligands and Top 15 PFAS bound to CAR. (A) 85532-75-8 (B) 1153-51-1 (C) 293754-55-9 (D) 662-28-2 (E) 306-91-2 (F) 307-08-4 (G) 118914-94-6 (H) 558-64-5 (I) 118914-93-5 (J) 97571-69-2 (K) 33021-47-5 (L) 306-99-0 (M) 118945-64-5 (N) 307-07-3 (O) 105462-77-9 (P) 125061-94-1 (Q) 61855-74-1 (R) 119107-96-9

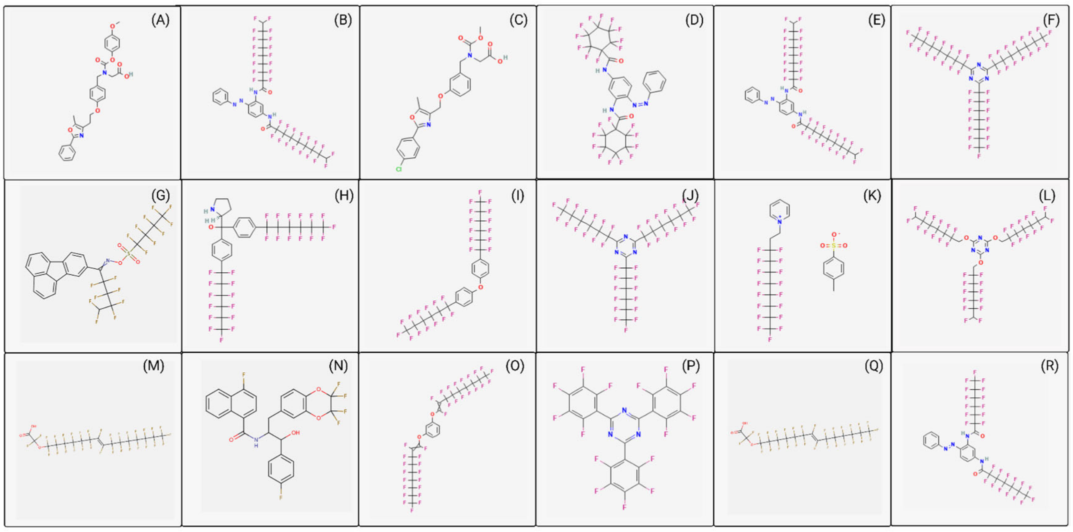

Supplementary Figure S19. 2D Structures of the Top 3 Endogenous Ligands and Top 15 PFAS bound to PPARα.
(A) 331741-94-7 (B) 1000998-62-8 (C) 1000998-59-3 (D) 548470-06-0 (E) 547748-28-7 (F) 21674-38-4 (G) NOCAS_1027003 (H) 1075687-38-5 (I) 151707-03-8 (J) 25761-65-3 (K) 61798-68-3 (L) 464-34-6 (M) NOCAS_1035154 (N) NOCAS_1026885 (O) 55987-23-0 (P) 160248-96-4 (Q) NOCAS_1035147 (R) 548455-52-3

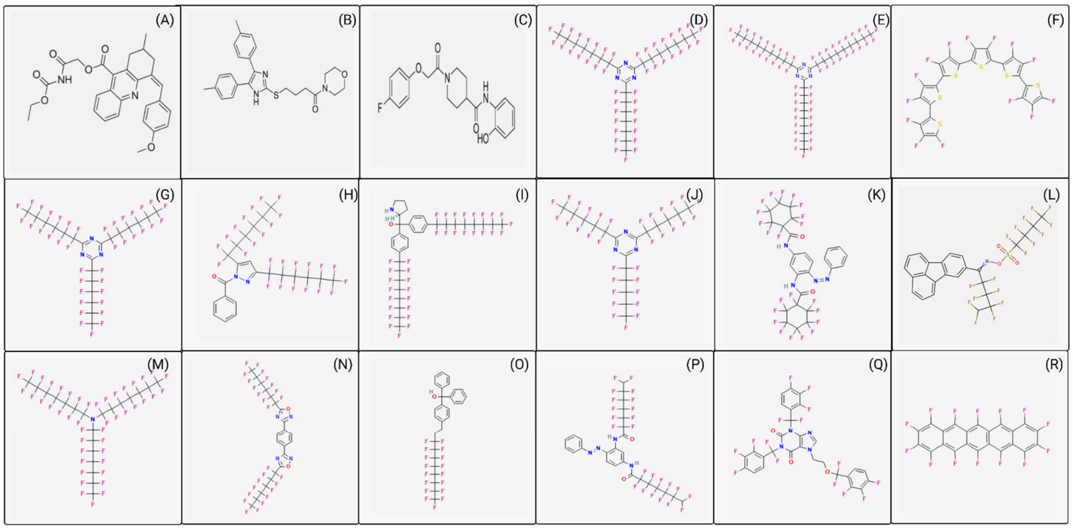

Supplementary Figure S20. 2D Structures of the Top 3 Endogenous Ligands and Top 15 PFAS bound to PPARδ. (A) GNF-8560 (B) GNF-9820 (C) GNF-5891 (D) 21674-38-4 (E) 57101-59-4 (F) 347838-21-5 (G) 25761-65-3 (H) 230295-10-0 (I) 862589-19-3 (J) 23790-50-3 (K) 548470-06-0 (L) NOCAS_1027003 (M) 1127427-75-1 (N) 146304-71-4 (O) 649561-66-0 (P) 548434-15-7 (Q) 126565-13-7 (R) 646533-88-2

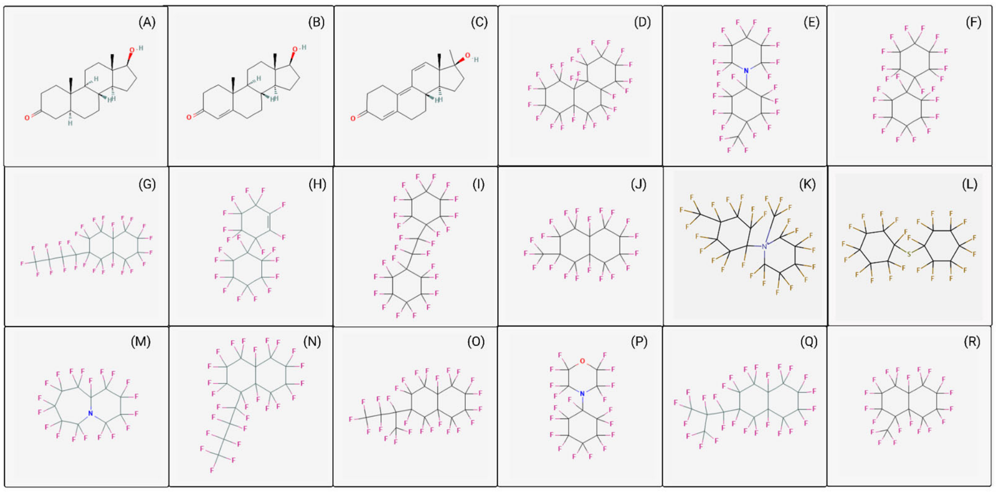

Supplementary Figure S21. 2D Structures of the Top 3 Endogenous Ligands and Top 15 PFAS bound to AR. (A) 521-18-6 (B) 58-22-0 (C) 965-93-5 (D) 306-91-2 (E) 86630-50-4 (F) 558-64-5 (G) 119107-96-9 (H) 61855-74-1 (I) 306-99-0 (J) 306-95-6 (K) NOCAS_1027599 (L) NOCAS_1026765 (M) 95827-25-1 (N) 118914-93-5 (O) 119141-86-5 (P) 114832-09-6 (Q) 119141-87-6 (R) 306-92-3

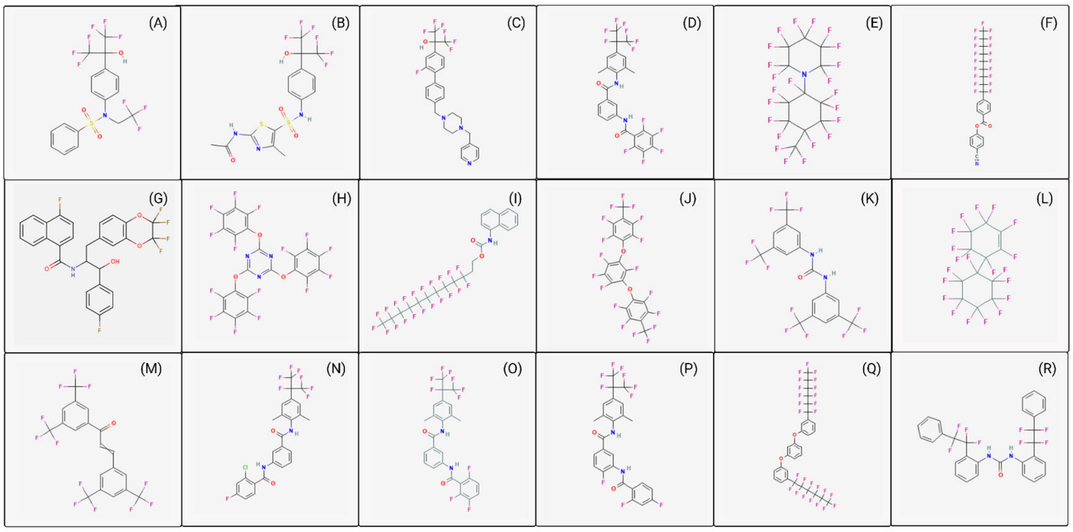

Supplementary Figure S22. 2D Structures of the Top 3 Endogenous Ligands and Top 15 PFAS bound to RORγt. (A) 293754-55-9 (B) 1335106-03-0 (C) 1359164-11-6 (D) 862130-96-9 (E) 86630-50-4 (F) 88951-08-0 (G) NOCAS_1026885 (H) 2663-96-9 (I) 305849-27-8 (J) 15038-90-1 (K) 3824-74-6 (L) 61855-74-1 (M) 819792-77-3 (N) 862130-87-8 (O) 862130-95-8 (P) 862133-29-7 (Q) 151707-04-9 (R) 61547-75-9

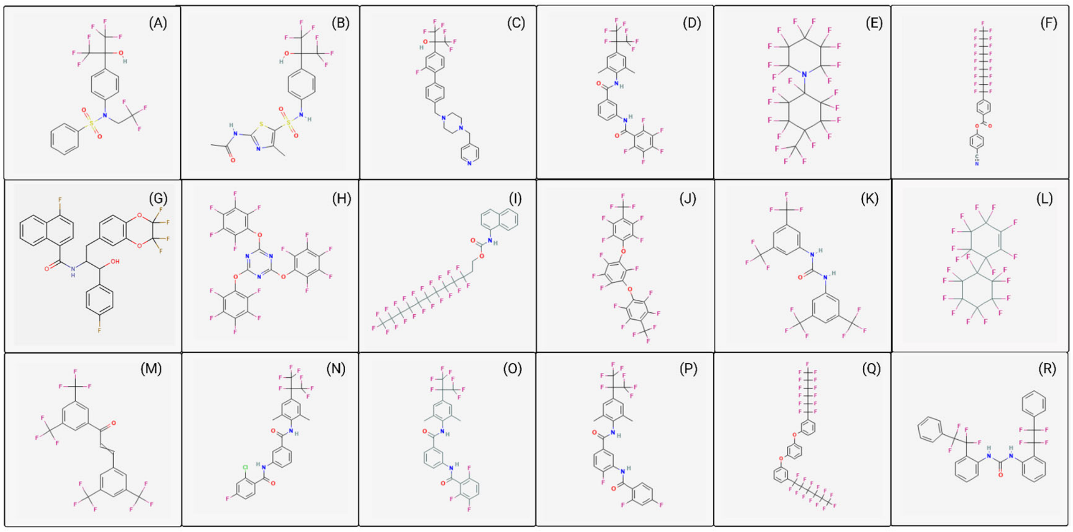

Supplementary Figure S23. 2D Structures of the Top 15 PFAS bound to RORγt. (A) 293754-55-9 (B) 1335106-03-0 (C) 1359164-11-6 (D) 862130-96-9 (E) 86630-50-4 (F) 88951-08-0 (G) NOCAS_1026885 (H) 2663-96-9 (I) 305849-27-8 (J) 15038-90-1 (K) 3824-74-6 (L) 61855-74-1 (M) 819792-77-3 (N) 862130-87-8 (O) 862130-95-8 (P) 862133-29-7 (Q) 151707-04-9 (R) 61547-75-9
